# Harnessing Acoustic Cavitation Energy for the Selective Degradation of Impurities in Glucose Syrup

**DOI:** 10.1002/chem.70992

**Published:** 2026-04-08

**Authors:** Shambel Getachew Wasse, Prince N. Amaniampong, François Jérôme

**Affiliations:** ^1^ CNRS Institut de Chimie des Milieux et Matériaux de Poitiers‐IC2MP Université de Poitiers Poitiers France

**Keywords:** biomass, cavitation bubbles, LogP, purification, ultrasound

## Abstract

Here, we open a path for cavitation bubbles to interact with a specific solute within a complex mixture. This tailored interaction enables the selective transfer of acoustic energy to a targeted chemical, allowing its degradation, primarily through localized cracking and oxidative mechanisms, without degrading the other components of the mixture. As a proof of concept, we investigated the purification of glucose syrup as an industrially relevant case. In brief, we have exploited the polarity differences between glucose and its impurities to locally confine cavitation bubbles at the proximity of these impurities, thereby promoting their specific decomposition without causing any degradation of glucose. Throughout this study, LogP proved to be a robust descriptor for interpreting and rationalizing our results.

## Graphical abstract

The energy released during the implosion of acoustic cavitation bubbles can be harnessed and selectively transferred to a target solute, enabling its specific decomposition without degrading other solutes present in the solution. This proof of concept was demonstrated in the degradation of impurities present in trace amount in glucose syrup.

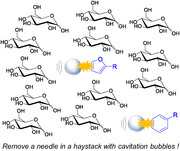

## 1 Introduction

The urgent need to move away from fossil energy has catalyzed the exploration of alternative, and ideally more sustainable, activation methodologies in chemical processes. For instance, activating chemicals using electrons [1, 2, 3], magnetic fields [4, 5], photons [6, 7, 8, 9], mechanical forces [10, 11, 12, 13, 14], and other approaches has become a major focus of current research. In this context, ultrasound is now witnessing a sort of renaissance [15, 16, 17, 18, 19]. In short, when piezoelectric materials are electrified, they vibrate and emit ultrasonic waves [20]. When these waves travel through a liquid such as water, the medium is subjected to compression and decompression phases [21, 22]. When the acoustic energy is high enough, it can disrupt the tensile strength of the liquid, forming cavitation bubbles that grow over time [23, 24]. Once these bubbles can no longer withstand the surrounding pressure, they collapse violently [25, 26]. The rapid compression of the gas inside the bubble at collapse time causes a transient localized increase in temperature and pressure [27, 28].

Recent work from our group [29, 30, 31] and others [15, 16, 17, 18, 19] has shown that the energy released during bubble implosion can be harnessed to activate and convert various organic molecules in water. Under certain conditions, molecules can even undergo pyrolysis to gaseous products at the bubble periphery, induced mainly by thermal and oxidative reactions. So far, most of existing studies focused on the ultrasonic activation and conversion of a single organic solute in water [15, 16, 17, 18, 19, 20, 21, 22, 23, 24, 25, 26, 27, 28, 29, 30, 31]. In contrast, the selective decomposition of a target molecule within a cocktail of solutes remains largely unexplored. This task is indeed considerably more challenging, as it requires the precise localization of cavitation bubbles at the proximity of the target solute only, to prevent degradation of other solutes in solution. To address this scientific question, we leveraged the ability of hydrophobic molecules to adsorb at the gas‐liquid interface [32, 33, 34]. This physical phenomenon is primarily governed by van der Waals interactions (such as dipole‐induced dipole forces and London dispersion forces), a phenomenon driven by a decrease in the system's Gibbs free energy (entropically favorable) [35]. Furthermore, the adsorption of hydrophobic molecules at the interface locally lowers the surface tension of water, in accordance with the Young–Laplace law, thereby promoting the growth of cavitation bubbles [36, 37, 38]. By exploiting the differences in solute hydrophobicity, we thus developed a method that selectively confines cavitation bubbles near the most hydrophobic solute of the mixture. This approach enables localized degradation of the target solute while preventing the degradation of other solutes in solution.

As a proof of concept, we investigated the purification of glucose syrup as an industrially relevant case study. During glucose extraction from biomass waste, impurities such as furfural, 5‐hydroxymethylfurfural (HMF), and various aromatic compounds are formed [39, 40]. Even at trace concentrations, these species can rapidly deactivate catalysts [41] or microorganisms involved in glucose‐based downstream processes [42, 43]. As a result, the degradation of these impurities is a mandatory step in biorefineries to ensure optimal performance in subsequent glucose process stages [44, 45, 46]. In this context, a variety of techniques have been implemented at both pilot and industrial scales, including recrystallization [47], filtration over activated charcoal or ion‐exchange resins [48, 49], nanofiltration using membranes [50, 51], selective precipitation [52], liquid–liquid extraction [53, 54], distillation [55], and bacterial decomposition [56, 57]. While these methods effectively degrade impurity levels, they also present drawbacks, such as the generation of aqueous waste and/or salt accumulation resulting from pH adjustments, for some of them, non‐negligible losses of glucose [58, 59]. In addition, these purification technologies are often limited in their ability to decompose impurities present in trace amounts [60], which today represents a significant large‐scale constraint and an area where innovation is expected.

## 2 Results and Discussion

**FIGURE 1:**
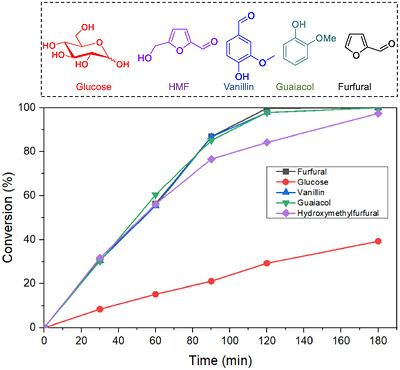
Conversion of aqueous solution of glucose and its common impurities over time under high frequency ultrasound (Ar flow‐30 mL/min, 35°C, 545 kHz, 1 mM each).

In an initial series of experiments, we examined the degradation of three representative impurities commonly present in glucose: furfural, 5‐hydroxymethylfurfural and guaiacol. Acoustic cavitation bubbles were formed by irradiating the aqueous solution of impurity (1 mM) at a high ultrasonic frequency (545 kHz). Unlike conventional low‐frequency ultrasound (< 100 kHz), which primarily induces physical effects (*e.g*., shock waves) due to the formation of large cavitation bubbles, high‐frequency ultrasound (> 100 kHz) has been preferred in this work as it produces a greater density of cavitation bubbles, and with a smaller size, thereby enhancing chemical effects (*e.g*., radical formation, thermal, and plasma‐like reactions) [61, 62, 63, 64]. The ultrasonic reactor was equipped with a cooling jacket to maintain the reaction volume (100 mL) at a constant temperature of 35°C (Figure S1). Argon gas was continuously bubbled through the solution at a flow rate of 30 mL/min throughout the reaction. The initial rates of conversion of all impurities were determined using HPLC (Figure S2). Further details regarding the ultrasonic reactor setup and analytical procedures are available in the Supporting Information. The results are summarized in Figure 1.

In our ultrasonic conditions, all tested impurities were independently converted at a similar rate of ∼ 0.6 mmol/L/h. Depending where the reaction is taking place, two different kinds of mechanism can be distinguished under high frequency ultrasound; (1) at the cavitation bubble interface where solutes are converted either by thermal cracking (the temperature at the gas‐liquid boundary can reach several hundred of degrees Celsius) [65, 66, 67] and by oxidative degradation thanks to ·OH radicals generated by the sonolysis of water inside cavitation bubbles (·H radicals are, for their part, recombined into H_2_.) [66, 68, 69] and (2) in the bulk solution where solutes are converted by ·OH radicals diffusing into the water phase. Overall, reactions occurring at the cavitation bubble‐water interface are significantly faster than those in the bulk solution because of the higher local energy and greater local concentration of ·OH radicals. To tentatively differentiate where the reaction is taking place, DMPO was added into the solution as a ·OH radical scavenger [70, 71]. Due to its hydrophilic nature, DMPO is expected to interact more weakly with the cavitation bubble‐water interface than vanillin, guaiacol, furfural, or HMF, which are more hydrophobic, at least when DMPO is present at a similar concentration. More evidences on this aspect are provided later. Therefore, DMPO appears as a potential probe molecule to determine whether the reactions occur with ·OH radicals diffusing into the solution or at the cavitation bubble periphery. Interestingly, adding DMPO, even in a fivefold excess (5 mM), did not completely inhibit the conversion of guaiacol, furfural, and HMF, it only decreased their initial rate of conversions by 55%, 70%, and 90%, respectively (Figures S3–S5). This observation suggests that these impurities are more likely converted at the cavitation bubble‐water interface, a result that aligns with our previous findings on the demethylation of *N*‐substituted aniline derivatives and anisole‐like compounds [29].

**SCHEME 1:**
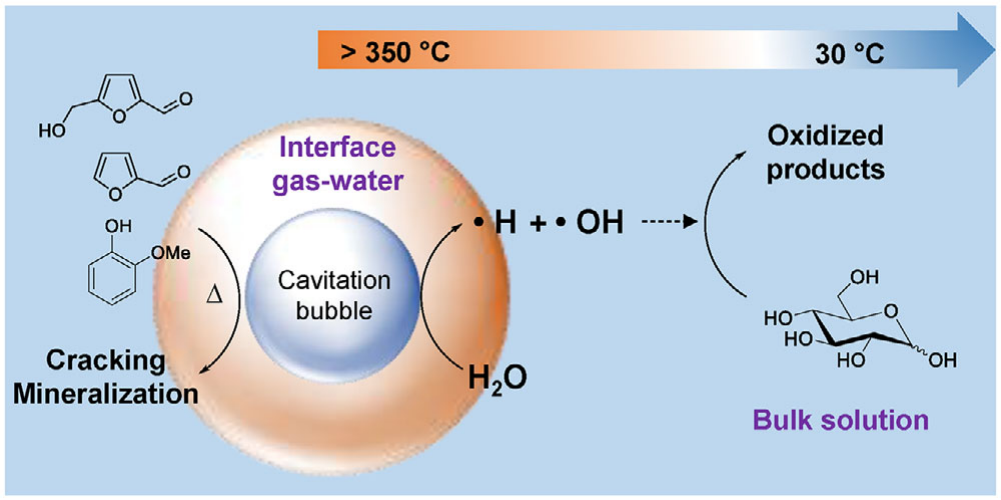
Reaction at the cavitation bubble‐water interface *vs* ^·^OH mediated oxidation reaction into the bulk water.

To assess the ability of cavitation bubbles to selectively decompose impurities from glucose aqueous solutions, *i. e*. without degrading glucose, a 1 mM solution of glucose was treated by high frequency ultrasound in similar conditions. As expected, because of its hydrophilic nature (Log P = −3.22), glucose weakly interact at the cavitation bubbles‐water interface. This is illustrated by an initial rate of conversion of glucose about 15 times slower than that of impurities (Figure 1). In contrast to tested impurities, addition of 5 mM DMPO completely quenches the conversion of glucose (Figure S6). This result confirms that glucose does not directly interact with cavitation bubbles but reacts more likely into the bulk water solution with diffusing ·OH radicals (Scheme 1).

**FIGURE 2:**
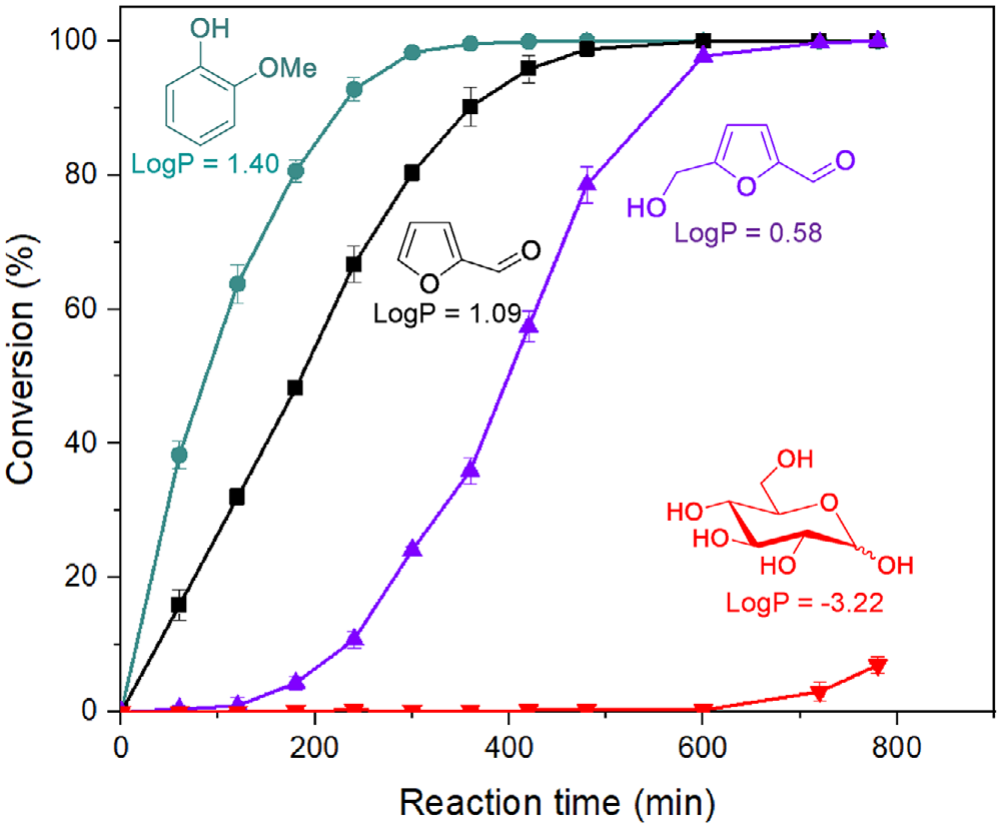
Conversion of a mixture of glucose with its common impurities over time under high frequency ultrasound (Ar flow‐30 mL/min, 35°C, 545 kHz, 1 mM each).

With these results in hand, mixtures of glucose and various impurities were subjected to ultrasonic irradiation to assess if this technology could purify selectively aqueous solution of glucose. In an initial experiment, a mixture containing 1 mM each of glucose, guaiacol, furfural, and HMF was treated with ultrasonic irradiation (Figure 2). Remarkably, guaiacol, furfural, and HMF were selectively decomposed without any degradation of glucose. More information on the nature of products formed are provided later. Interestingly, while guaiacol, furfural, and HMF were converted at similar rates when tested individually (Figure 1), these three impurities were not reacted simultaneously when combined together with glucose in a single aqueous mixture (Figure 2). In this scenario, although their degradation rate remained similar, guaiacol was converted first, followed by furfural and HMF. Interestingly, HMF only began to degrade after guaiacol was fully converted and approximately 85% of furfural had been degraded. Throughout this period, glucose remained unreacted. Glucose degradation only began once HMF had reached around 90% conversion. Overall, and consistent with the work of Okitsu [72], the reactivity order of these compounds appears to correlate with their LogP values (a measure of hydrophobicity): guaiacol (LogP = 1.40) > furfural (LogP = 1.09) > HMF (LogP = 0.58) > glucose (LogP = −3.22). In other words, the higher the LogP value, the sooner the compound is converted by cavitation bubbles, providing further experimental evidence of the strong interaction of hydrophobic molecules at the cavitation bubble‐water interface.

From a practical standpoint, this result is highly encouraging, as it demonstrates that in a mixture of compounds: (1) the most hydrophobic molecules are converted first, and more importantly, (2) as long as the most hydrophobic substrate remains in the solution, it effectively protects the others from degradation (probably by trapping the heat and radicals generated at the cavitation bubble collapse time).

To assess the fate of impurities decomposed by cavitation bubbles, the carbon concentration in the liquid phase was monitored over time using a Total Organic Carbon (TOC) analyzer. After 2 h of ultrasonic treatment, 35% of the converted carbon consist of gaseous products, consistent with the thermal cracking of impurities. Real‐time monitoring of the reactor headspace by online gas chromatography during the reaction confirmed the formation of gaseous products, predominantly methane, carbon dioxide, ethylene, and acetylene, typical markers of thermal cracking, with concentrations increasing over time (Table S1). Hydrogen evolution was also detected, with 41% of this evolution originating from water sonolysis and 59% from impurity cracking. To gain further insight into the nature of water‐soluble products formed, the liquid phase was analyzed by NMR (with reactions conducted in D_2_O) and by HPLC. In the ^1^H NMR spectra, beside glucose and unreacted impurities, only apparition of an additional singlet at 8.2 ppm was observed during ultrasonic irradiation, and corresponding to formic acid (Figure S7). This assignment was corroborated by HPLC analysis, which revealed a product with a retention time comparable to that of formic acid (Figure S8). Taken together, these results indicate that, at the cavitation bubble‐water interface, impurities are predominantly cracked into gaseous products and/or mineralized to formic acid, which itself is further converted *in situ* to CO_2_ over time, as supported by gas‐phase analysis. At this stage, the formation of other soluble compounds at concentrations below the detection limits of our analytical apparatus cannot be excluded, likely including oxidized intermediates (*e.g*., carboxylic acids), as suggested by a slight decrease in solution pH from 6.5 to 4. To support this claim, the aqueous phase was analyzed by LC‐HRMS which offer a higher sensitivity. As expected, beside formic acid, a cocktail of oxygenated products, mainly C3‐C6 products, was detected, further supporting the partial mineralization of sugar syrup impurities (Table S2). In some cases, particularly at the early stage of ultrasonic irradiation, an additional singlet at 3.2 ppm was observed in the ^1^H NMR spectrum (Figure S7). This signal may be tentatively attributed to dimethyl ether, a compound formed *via* the dimerization of methanol, which is a primary product observed during the thermal cracking of guaiacol. We also quantified the formation of H_2_O_2_, which can result from the recombination of ·OH radicals. Interestingly, while the initial H_2_O_2_ formation rate was 0.5 mmol·L^−^^1^·h^−^^1^ in pure water, it decreased to only 0.08 mmol·L^−^^1^·h^−^^1^ during ultrasonic treatment of the aqueous solution containing guaiacol, furfural, HMF, and glucose (Figure S9). This observation is consistent with our results and confirms that impurities strongly interact at the cavitation bubble‐water interface, where they also trap ·OH radicals to be mineralized to formic acid and CO_2_. Note that, in many cases, carboxylic acids do not pose a problem for downstream glucose conversion, as these steps mainly involve acid catalysts, even including sometimes formic acid. For downstream enzymatic processes, the conventional use of buffers also mitigates potential deactivation of enzymes or microorganisms by formic acid.

**FIGURE 3:**
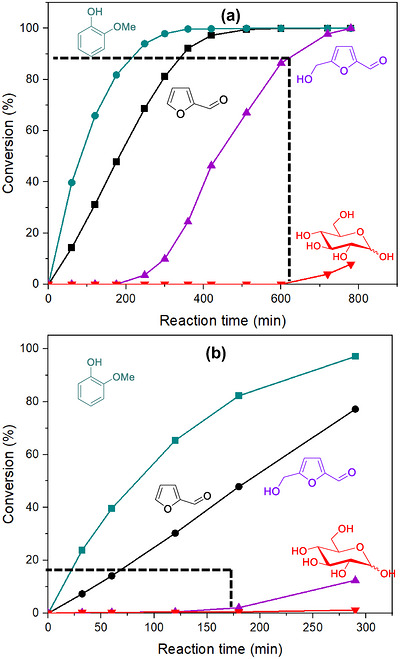
Conversion of a mixture of glucose with common impurities over time under high frequency ultrasound (a) under Ar and (b) under Ar/O_2_ (80/20) (gas flow‐30 mL/min, 35°C, 545 kHz, 1 mM each).

While modifying the water temperature of the bulk aqueous solution of glucose (from 10°C to 35°C) had no significant impact on the process efficiency, it was not the case of the gaseous atmosphere, in particular when oxygen (or air) was introduced into the ultrasonic reactor (Figure 3). For example, the initial rate of conversion of impurities (guaiacol, furfural, and HMF) was found to be 30% higher under an Ar/O_2_ (2:1) atmosphere compared to pure Ar. This conversion rate improvement can be attributed to differences in gases specific heat capacities, thermal conductivities, and solubilities in water but, more likely to a higher production of oxygenated radicals (·OH and ·OOH) in the presence of oxygen. This hypothesis was further supported by quantifying the amount of H_2_O_2_ generated in our reactor during high‐frequency ultrasonic irradiation of pure water (*i.e*. no chemical was introduced). The H_2_O_2_ formation rate was found to be 3.5‐fold higher under an Ar/O_2_ atmosphere compared to Ar alone (Figure S10). In line with this conclusion, the process was found to be less selective under Ar/O_2_ than under pure Ar. Indeed, under Ar/O_2_, while guaiacol and furfural were still selectively degraded without affecting glucose, this was not the case anymore for HMF. Under Ar, glucose degradation began only when HMF conversion exceeded 80%, whereas under Ar/O_2_, this conversion threshold dropped to 15%. This loss in selectivity observed in the presence of O_2_ (or air) is primarily attributed to the overproduction of oxygenated radicals during cavitation bubble collapse, which then diffuse into the bulk solution and partially oxidize glucose. In addition, while under Ar, nearly no product was detected by HPLC, under Ar/O_2_, a much more complex mixture of different products, presumably carboxylic acids or alkyl alcohols considering our HPLC column (COREGEL 107H), were detected as intermediates in the liquid phase. This observation clearly indicates the occurrence of concurrent reactions (*e.g*. sono‐Fenton‐like process) within the bulk liquid phase (Figure S11).

**FIGURE 4:**
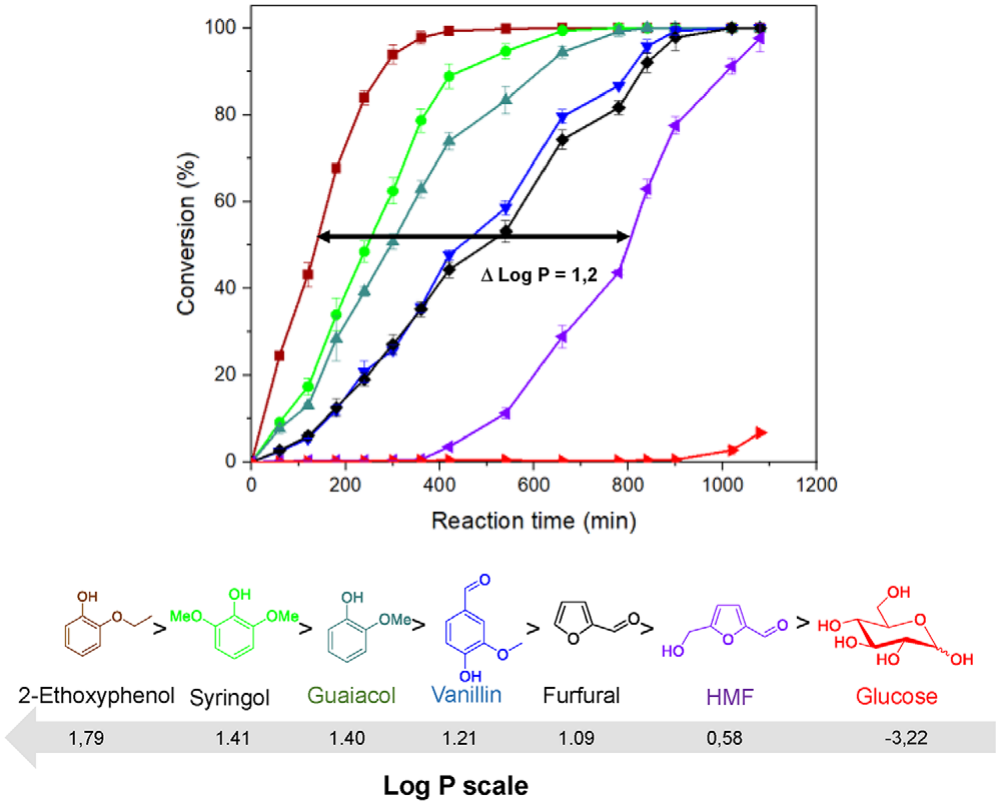
Conversion of a complex mixture of glucose with common impurities over time under high frequency ultrasound‐Correlation of the reactivity with the LogP (Ar flow‐30 mL/min, 35°C, 545 kHz, 1 mM each).

Encouraged by these findings, we proceeded to purify more complex and representative mixtures commonly encountered in the sugar industry, using our above‐described optimized conditions (30 mL/min Ar, 35°C, 545 kHz). For this purpose, glucose was combined with a cocktail of impurities, including furfural, HMF, guaiacol, 2‐ethoxyphenol, syringol, and vanillin, each at a concentration of 1 mM. In these experiments, the glucose concentration was also set at 1 mM to evaluate the selectivity of this technology. To our satisfaction, all the impurities were successfully decomposed from the aqueous glucose solution without any degradation of the glucose itself (Figure 4).

**FIGURE 5:**
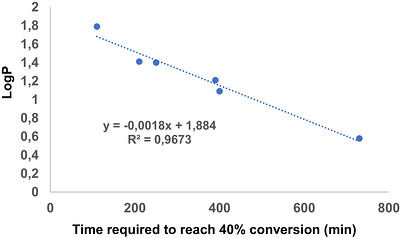
Plot of the LogP against the time required to reach 40% conversion for each impurity (Ar flow‐30 mL/min, 35°C, 545 kHz, 1 mM each).

Once again, the reactivity order closely followed the LogP values of the mixture components: 2‐ethoxyphenol (LogP = 1.79) was converted first, followed by syringol (LogP = 1.41), guaiacol (LogP = 1.40), vanillin (LogP = 1.21), furfural (LogP = 1.09), HMF (LogP = 0.58), and finally glucose (LogP = −3.22). A linear correlation was even observed when the LogP was plotted against the time required to reach 40% conversion for each impurity (Figure 5). Regardless of the number of impurities present with glucose (6, 5, 4, or 3), the slope of this linear correlation remains approximately –0.002 (Figure S12), further supporting the linear correlation between the time and the LogP. Consistent with this finding, a satisfactory mathematical model was developed that closely reproduces the experimental kinetic profiles shown in Figure 4. This mathematical model, described in the Supporting Information (Figure S13), depends on only two variables: time and the LogP value. Taken together, these correlations demonstrates that LogP is a reliable descriptor for predicting the order of reactivity of impurities in aqueous glucose solutions. Note that the LogP of glucose does not fit these derived equations, further confirming that its conversion is not governed by interactions with cavitation bubbles (*i.e*. by LogP). This observation is consistent with the previously proposed alternative pathway involving ·OH radicals for sonochemical conversion of glucose. As a general trend, this selective ultrasonic‐assisted purification technology is applicable for chemicals having a LogP value > 0.5. In sonochemistry, direct evidence of reaction taking place at the cavitation bubble‐water interface using an independent diagnostic or sensitivity analysis is unfortunately extremely challenging, mainly due chaotic and extremely dynamic nature of cavitation systems, and also due to the extreme transient pressure and temperature conditions prevailing at the time of bubble collapse, as well as the very short lifetime of cavitation events. Therefore, the LogP value offers indirect evidence for distinguishing between thermal cracking reaction at the cavitation bubble‐water interface and oxidation reactions occurring into the bulk solution.

Given that all impurities exhibit similar rate of conversions under ultrasonic conditions, it becomes also possible to estimate the required difference in LogP between two solutes to selectively decompose one compound without affecting the other. For example, it takes approximately 400 min to completely decompose 2‐ethoxyphenol (LogP = 1.79). By applying this value to our correlation equation, it can be deduced that a LogP difference of about 1.16 relative to 2‐ethoxyphenol should be sufficient to achieve the selective decomposition of 2‐ethoxyphenol from compounds with LogP values below 0.63.

To support this claim, two aqueous mixtures were prepared: one containing 1 mM guaiacol (LogP = 1.40) and 1 mM HMF (LogP = 0.58), corresponding to a ΔLogP of 0.82, and the other containing 1 mM 2‐ethoxyphenol (LogP = 1.79) and 1 mM HMF (ΔLogP = 1.21). Both mixtures were subjected to ultrasonic irradiation. As expected, HMF began to convert once guaiacol had reached approximately 60% conversion, whereas this threshold increased to about 90% when HMF was mixed with 2‐ethoxyphenol (Figures S14, 15). This finding strongly supports the idea that LogP could serve as a predictive descriptor of solute reactivity in complex mixtures exposed to high‐frequency ultrasonic irradiation.

To further assess the performance of this purification pathway, the concentration of HMF was gradually increased from 1 to 100 mM in a mixture containing 1 mM of 2‐ethoxyphenol. Interestingly, even with a 100‐fold excess of HMF, 2‐ethoxyphenol was still selectively converted (Figure S16). At this ΔLogP = 1.21, increasing the HMF concentration from 1 to 10 and 100 mM only reduced the initial rate of conversion of 2‐ethoxyphenol by ∼ 10% and ∼30%, respectively, demonstrating the preferential interaction of cavitation bubbles with the most hydrophobic molecules (Figure S17). Although limited, the partial competitive co‐adsorption of HMF at the cavitation bubble interface at high HMF/2‐ethoxyphenol molar ratios shifts the onset of HMF conversion, that is, at higher HMF concentrations, its conversion begins earlier (Figure S16). For example, at HMF concentrations of 1 and 10 mM, HMF started to convert only after 2‐ethoxyphenol had reached approximately 90% and 50% conversion, respectively. However, when the ΔLogP is larger, which is the typical case between glucose and its impurities, the selectivity of cavitation bubbles for the most hydrophobic molecules can be maintained even at high concentration of glucose. For instance, when 1 mM of 2‐ethoxyphenol was mixed with increasing concentrations of glucose (1–100 mM) and exposed to high‐frequency ultrasonic irradiation, 2‐ethoxyphenol was converted at a similar rate, indicating the absence of competitive adsorption between 2‐ethoxyphenol and glucose at the cavitation bubble interface (Figure S18). As a result, 2‐ethoxyphenol can still be selectively decomposed from more concentrated glucose solutions without causing any glucose degradation.

Having all these results in our hands, this technology was transposed to a more industrially realistic sugar juice. To this end, the glucose concentration was increased to 10 wt%, while keeping the concentration of impurities (guaiacol, furfural, HMF) at 1 mM. Even at this elevated glucose concentration, degradation of impurities was achieved without degrading glucose (Figure S19). The initial rate of conversion of all these impurities were similar regardless of the tested glucose concentrations (up to at least 10 wt%), further demonstrating the selective affinity of cavitation bubbles for hydrophobic molecules (Table S3). Overall, these results further demonstrate that as long as the LogP difference remains sufficiently large, cavitation bubbles can selectively decompose trace‐level impurities from glucose syrups, a notable advantage over existing purification technologies.

To evaluate the versatility of this technology, the method was further tested on other monosaccharides such as xylose (LogP = −2.74), mannose (LogP = −3.22), and fructose (LogP = −3.38), as well as on disaccharides such as maltose (LogP = −5.40) and sucrose (LogP = −5.40), each containing 1 mM guaiacol, 1 mM furfural, and 1 mM HMF. Consistent with the results obtained for glucose, all these impurities were selectively degraded in these aqueous sugar solutions (Figures S20–S24). Notably, regardless of the starting sugar, guaiacol, furfural, and HMF were converted in the same order and at comparable rates, confirming once again the specificity of cavitation bubbles toward these sugar impurities. As with glucose, increasing the xylose concentration to 10 wt% did not affect the selectivity of cavitation bubbles, that is, impurities were still degraded without causing any degradation of xylose (Figure S25).

To assess the energy efficiency of this ultrasonic technology, we applied it to the purification of a sugar juice produced by the acid‐catalyzed hydrolysis of sucrose into glucose and fructose. This reaction was selected because it is an industrially implemented process for producing monomeric sugars from sugar‐beet or sugar cane [73, 74]. At large scale, this reaction is typically carried out using concentrated sucrose solutions (30 wt%) (Supporting Information). At such high concentrations, side reactions can lead to the formation of HMF and its recombination products with sugars, commonly referred to as humins. Even in trace amounts, these impurities are known to deactivate catalyst (*e.g*. cocking) in glucose‐based downstream processes.

**FIGURE 6:**
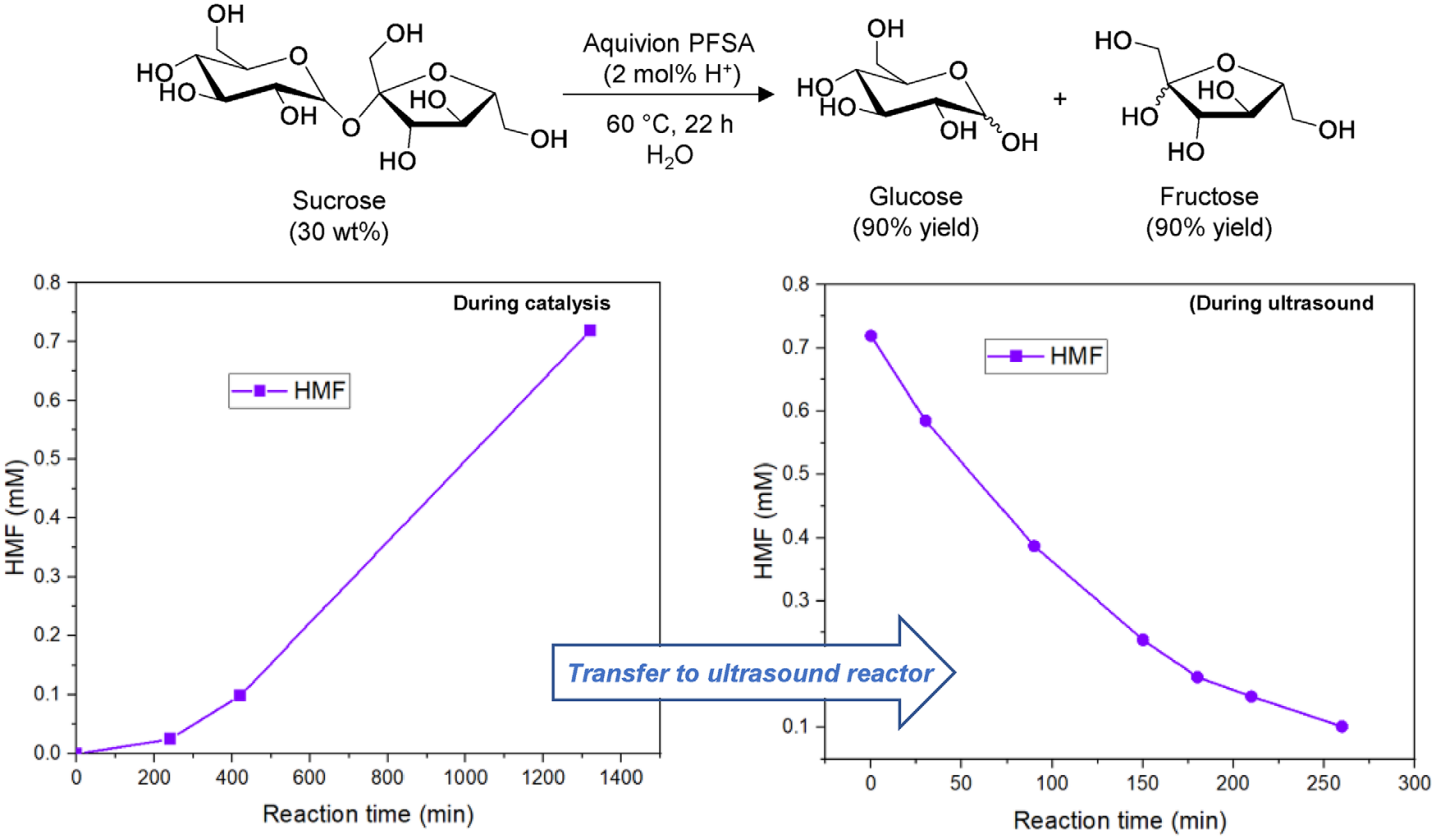
(A) formation of trace amounts of HMF during acid‐catalyzed sucrose hydrolysis and (B) sonochemically‐induced degradation of HMF from glucose‐fructose juice (conditions for ultrasonic treatments: under Ar at a gas flow rate of 30 mL/min, 35°C, 545 kHz).

In this context, a 30 wt% aqueous sucrose solution was heated to 60°C in the presence of Aquivion PFSA (perflurorinated sulfonic acid polymer) as a solid acid catalyst (2 mol% H^+^). The catalytic hydrolysis of sucrose and the concurrent formation of HMF were monitored by HPLC (Figures S26, S27). Under these conditions, glucose and fructose were obtained with a 90% yield after 22 h of reaction (Figure 6). As expected, a trace amount of HMF (0.7 mM) was also detected in the solution (Figure 6A). At the end of the reaction, Aquivion PFSA was separated from the liquid phase by filtration and the as‐collected sugar juice was treated by ultrasonic irradiation (545 kHz, 30°C, 0.17 W/mL). To our delight, as previously observed in model feeds of aqueous sugar solutions, HMF was selectively decomposed without causing degradation of glucose and fructose (Figures 6B, S28 and S29). Although our batch ultrasonic reactor is not designed for industrial‐scale applications, it is possible to estimate the energy consumption of this technology and assess its potential application on a larger scale. In the experiments described above, calorimetric measurements indicated an acoustic power density of 0.157 W/mL. For a 100 mL volume, this corresponds to 15.7 W, or 15.7 Joules per second. Complete degradation of HMF from the sugar juice required 4 h of ultrasonic treatment, amounting to an energy consumption of 226 kJ to treat 30 g of sucrose, that is, 7.5 kJ/g of sucrose. The energy consumption target at an industrial scale is generally lower than 20 kJ/g of product (including both reaction and downstream processing), which further highlight the potential of this technology as a purification method [75]. We would like to aware readers that the calculated energy efficiency does not take into account the conversion of electric power to acoustic energy by piezoelectric materials, which is material and supplier dependent (see Supporting Information for more details). We opted for the acoustic power density to ensure accurate comparisons (and reproducibility) with existing literature.

In our previous studies [29, 30, 31], we demonstrated that energy consumption can be significantly reduced by switching from a batch to a microfluidic flow ultrasonic reactor or by employing cavitation‐assisting agents [76], In particular, in a recent studies, we have reported that the rate at which is converted a solute by acoustic cavitation bubbles is exponentially increasing with the acoustic power density (and conversely for energy consumption), indicating substantial opportunities for further improvements in energy efficiency and scale up [77].

## 3 Conclusion

In conclusion, we report the use of high‐frequency ultrasound as a promising alternative technology for glucose syrup purification. By leveraging the substantial polarity difference between glucose and its common impurities, we demonstrate a pathway for selectively transferring acoustic energy to these impurities, even present at trace levels, thereby enabling their specific degradation without inducing any decomposition of glucose. The LogP value was found as a reliable descriptor to interpret and rationalize our observed results. This technology has been then assessed on the hydrolysis of sucrose, an industrially deployed reaction, where we showed that the energy consumption was close to the industrial benchmarks of the field, thereby opening promising prospects for scale‐up. Compared with existing purification technologies, this approach stands out by enabling the selective degradation of impurities from glucose syrup (i) without glucose degradation, and (ii) without the need for catalysts. Moreover, the ultrasonic reactor operates at 35°C and atmospheric pressure, providing additional benefit in terms of safety.

Evaluation of this technology for the purification of sugar syrups prior to the bioconversion of carbohydrates into fine chemicals is now the topic of current investigations in our group, with a particular focus on enhancing microorganism performances. This study will be published separately as it requires to tackle additional scientific hurdles notably linked to the presence of proteins and carboxylic acids.

## Conflicts of Interest

The authors declare no conflicts of interest.

## Supporting information




**Supporting File 1**: chem70992‐sup‐0001‐SuppMat.docx.

## Data Availability

The data that support the findings of this study are available from the corresponding author upon reasonable request.
