# Supplementary material for: Harnessing Acoustic Cavitation Energy for the Selective Degradation of Impurities in Glucose Syrup
Source: Chemistry. 2026 Apr 8;32(23):e70992. doi: 10.1002/chem.70992 (PMC13282905; doi:10.1002/chem.70992)
Supplement: Supplementary file 1 — Supporting File 1: chem70992‐sup‐0001‐SuppMat.docx. [file CHEM-32-e70992-s001.docx]

**Supporting Information**

**Harnessing Acoustic Cavitation Energy for the Selective Degradation of Impurities in Glucose Syrup**

Shambel Getachew Wasse,^[a]^ Prince N. Amaniampong, ^[a]^ and François Jérôme*^[a]^

^[a]^ CNRS, Université de Poitiers, Institut de Chimie des Milieux et Matériaux de Poitiers-IC2MP, 1 rue Marcel Doré, 86073 Poitiers, France

**Ultrasonic reactor setup**

The ultrasonic reactor was acquired from SinapTec Ultrasonic Technology *(Fig. S1*) (ultrasonic generator NexTgen LAB1000). This reactor incorporates three piezoelectric materials that convert electricity (75 W) to acoustic power (15 W) (*i. e*. 20% efficiency), thus producing ultrasonic waves at a frequency of 545 kHz. During ultrasonic irradiation, piezoelectric materials were continually cooled by an airflow as shown in figure S1. The solution's temperature was monitored using a thermocouple and maintained at 30°C with a cooling jacket.


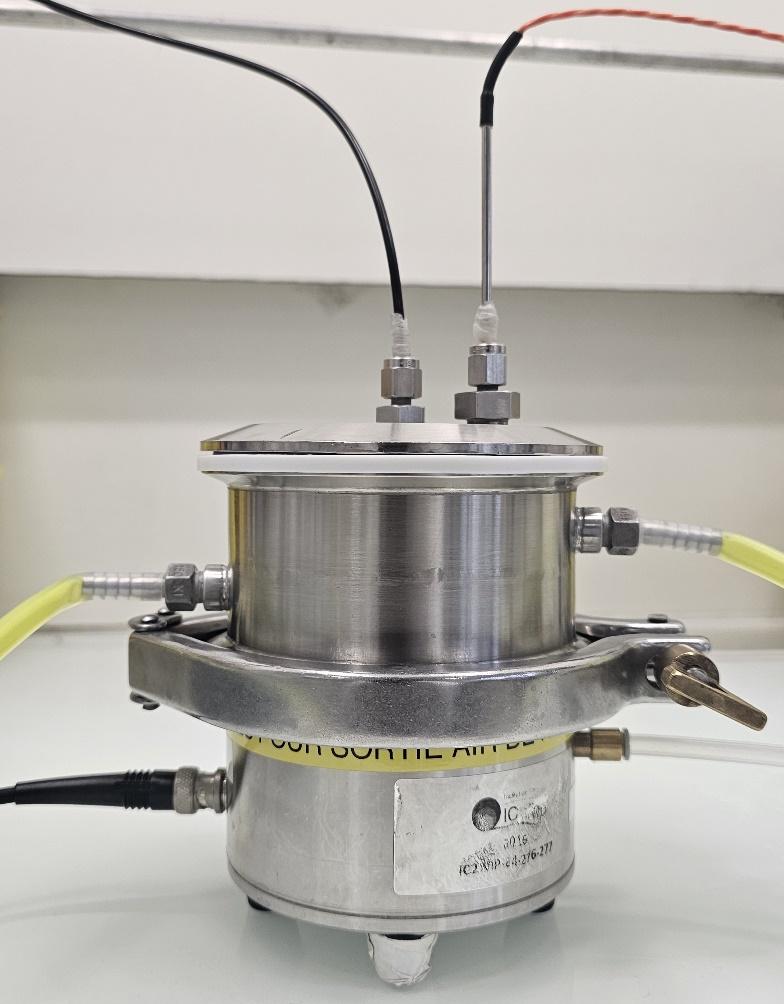


G

H

E

D

C

A

B

F

**Figure S1**. Ultrasonic reactor photo. A). Transducer (made up of piezoelectric materials) B) Power source C) Cooling oil inlet D) Gas bubble inlet E) Temperature controller F) Cooling oil outlet G) Cooling jacket H) Cooling air inlet to cool-down the transducer.

The acoustic power of the reactor was determined by a calorimetric method.^[1]^ The acoustic power was calculated by the formula P_ac_=m.C_P_.ΔT/Δt with P_ac_ is the acoustic power (W), m is the mass of liquid (kg), c_P_ is the specific heat capacity of the liquid (J.kg^-1^.K^-1^), ΔT is the temperature variation (°K) and Δt is the time variation (s). Calculations revealed an acoustic power density of 0.157 W/mL.

**General procedure**

In a typical experiment, a 100 mL aqueous solution containing 1 mM–10 wt% of glucose and 1 mM of an impurity (guaiacol, furfural, 5-hydroxymethylfurfural, vanillin, syringol, or 2-ethoxyphenol) was first degassed by continuously bubbling Ar at a flow rate of 30 mL/min for 20 min. The resulting solution was then subjected to ultrasonic irradiation (545 kHz) at 30 °C and at an acoustic power density of 0.157 W/mL, while maintaining Ar bubbling at 30 mL/min throughout the sonication. During the reaction, aliquots were taken and analyzed by HPLC to determine the conversion, yield, and selectivity.

**Note**: The same procedure was applied to other sugars (fructose, mannose, xylose, sucrose, maltose) or under different gas atmospheres (O₂ and air) described in the main article text.

**Procedure for acid-catalyzed hydrolysis of sucrose**

A 30 wt.% aqueous sucrose solution was prepared in a 100 mL volumetric flask and then transferred to a three-neck round-bottom flask equipped with a thermoregulator set to 60 °C. Subsequently, 2 g of Aquivion^®^ PFSA (a perfluorinated sulfonic acid polymer serving as a solid acid catalyst, 0.98 mmol H^+^/g) was added. The mixture was stirred at 600 rpm with a magnetic stirrer and allowed to react for 22 hours. The conversion of sucrose and the formation of the main products, glucose and fructose, as well as the side product HMF, were monitored by HPLC.

**H_2_O_2_ titration**

H_2_O_2_ titration was made with titanium oxysulfate [TiOSO_4_] under acidic condition. 0.55 g of [TiOSO_4_] was dissolved in water and then 2.8 mL of H_2_SO_4_ were added. The solution was completed with water to a final volume of 100 mL. The final concentration of TiOSO_4_ and H_2_SO_4_ became 0.02 M and 0.5 M respectively. For calibration, the hydrogen peroxide solutions were prepared from a commercial H_2_O_2_ solution (30 wt%), with concentration in the 0-0.002 mol/L range. The absorbance was measured at 412 nm.

**HPLC injection protocols**

Guaiacol, furfural, 5-hydroxymethylfurfural, vanillin, syringol and 2-ethoxyphenol were analyzed using high-performance liquid chromatography (HPLC) from SHIMADZU LC-40D equipped with a UV-Visible detector (225 nm) and a C18 Agilent column (4.6 mm × 250 mm). The eluent was composed of a mixture of acetonitrile/water (25:75) flowing through the column at a flow rate of 0.8 mL min^-1^. The injection volume was 20 μL, and the temperature of the column was set at 25 °C. For calibration, standard solutions of guaiacol, furfural, 5-hydroxymethylfurfural, vanillin, syringol and 2-ethoxyphenol were prepared in water, at concentrations ranging from 0 to 2 mM. Calibration curves are provided in Fig. S2 were used for the determination of conversion provided in the article text. Data were given with an uncertainty of ± 2%.

Sugars (glucose, mannose, fructose, xylose, maltose, sucrose) were analyzed by HPLC on a SHIMADZU CORP equipped with a RID detector and a NH2P-50 4E column*.* The eluent was composed of ultrapure pure water, containing 0.04 wt% of H_2_SO_4_, flowing through the column at a flow rate of 0.8 mL min^-1^. The injection volume was 20 μL, and the temperature of the column was set at 40 °C. For calibration, standard solutions of sugars were prepared in water, at concentrations ranging from 0.5 to 5 mM. Calibration curves are provided in Fig. S2 and were used for the determination of conversion provided in the article text. Data were given with an uncertainty of ± 3%.

|  |  |
| --- | --- |
|  |  |
|  |  |
|  |  |
|  | 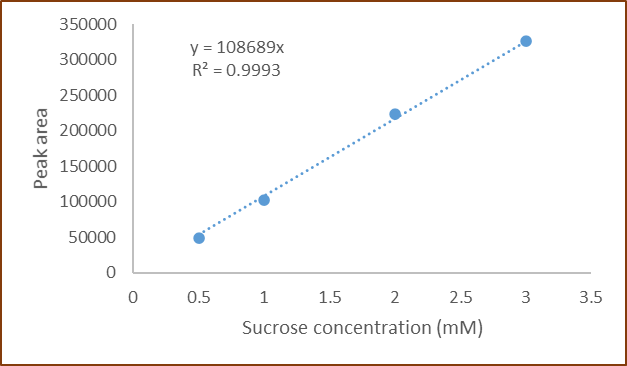 |
| 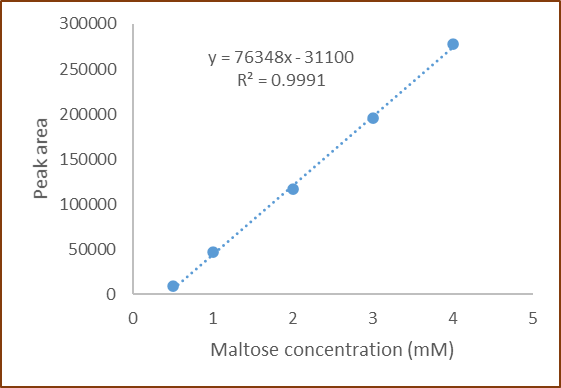 |  |
| 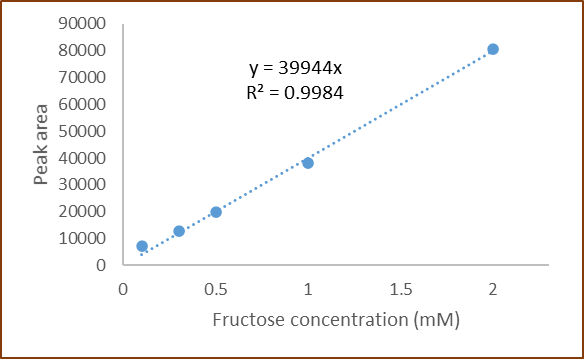 | 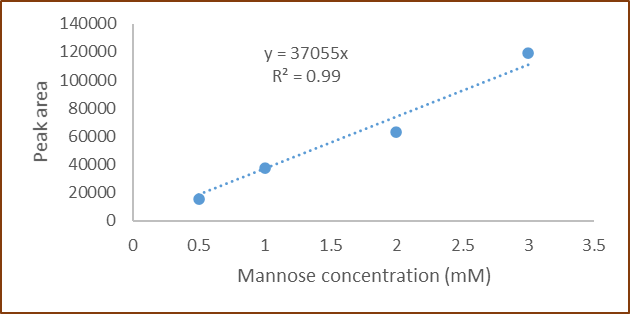 |

**Figure S2**. HPLC calibration curves of impurities and sugars used for plotting the conversion versus the ultrasonic reaction time


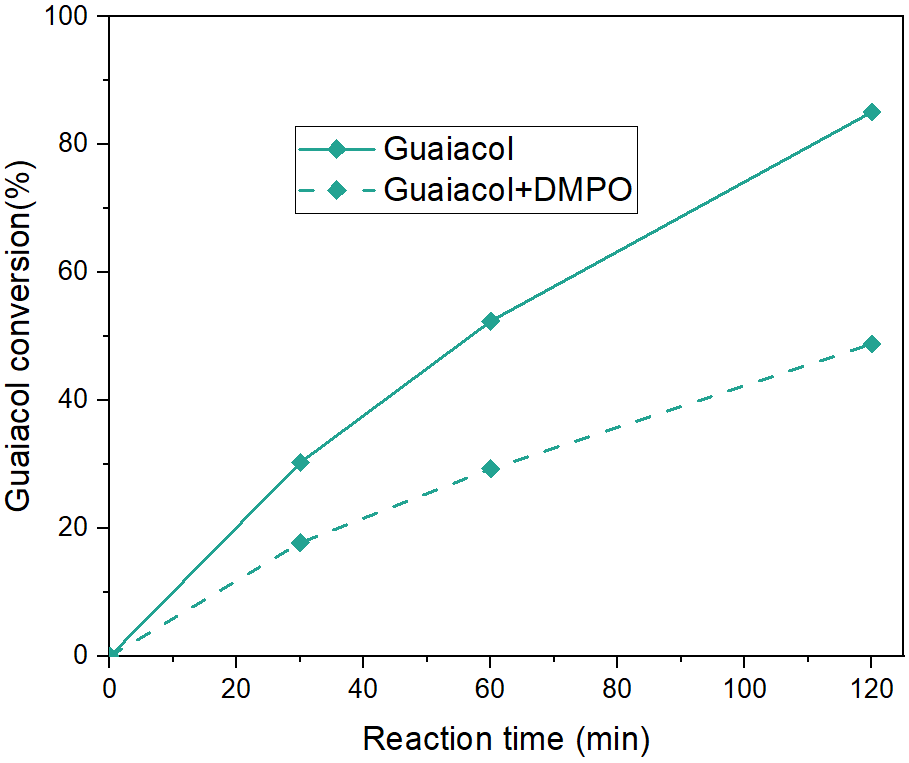


**Figure S3**. Guaiacol conversion with (dashed line) and without DMPO (plain line). Reaction conditions:1 mM of Guaiacol, 5 mM of DMPO, 100 mL of H_2_O, 35 °C, 545 kHz, Ar flow 30 mL/min.


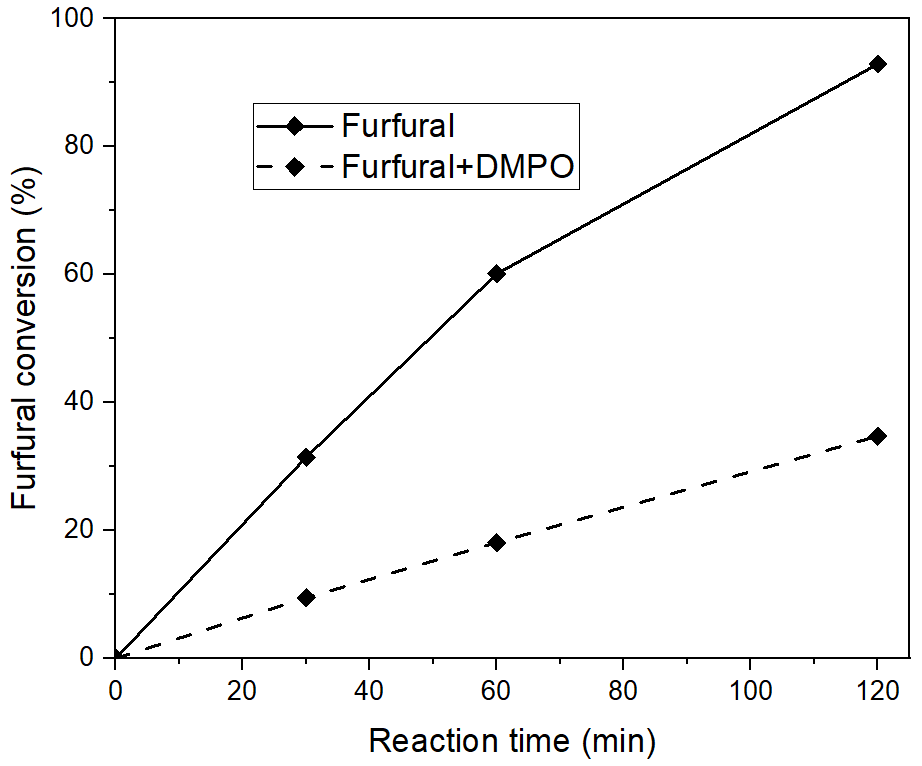


**Figure S4.**  Furfural conversion with (dashed line) and without DMPO (plain line). Reaction conditions: 1 mM of Furfural, 5 mM of DMPO, 100 mL of H_2_O, 35 ^°^C, 545 kHz, Ar flow 30 mL/min.


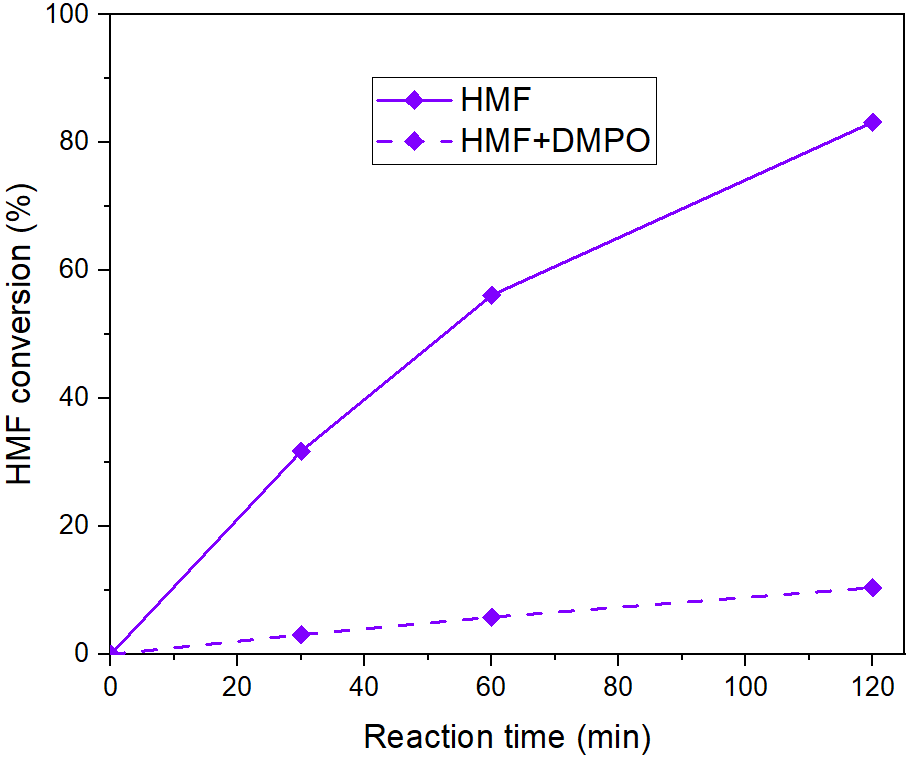


**Figure S5.** HMF conversion with (dashed line) and without DMPO (plain line). Reaction conditions: (1 mM of Furfural, 5 mM of DMPO, 100 mL of H_2_O, 35 ^°^C, 545 kHz, Ar flow 30 mL/min).


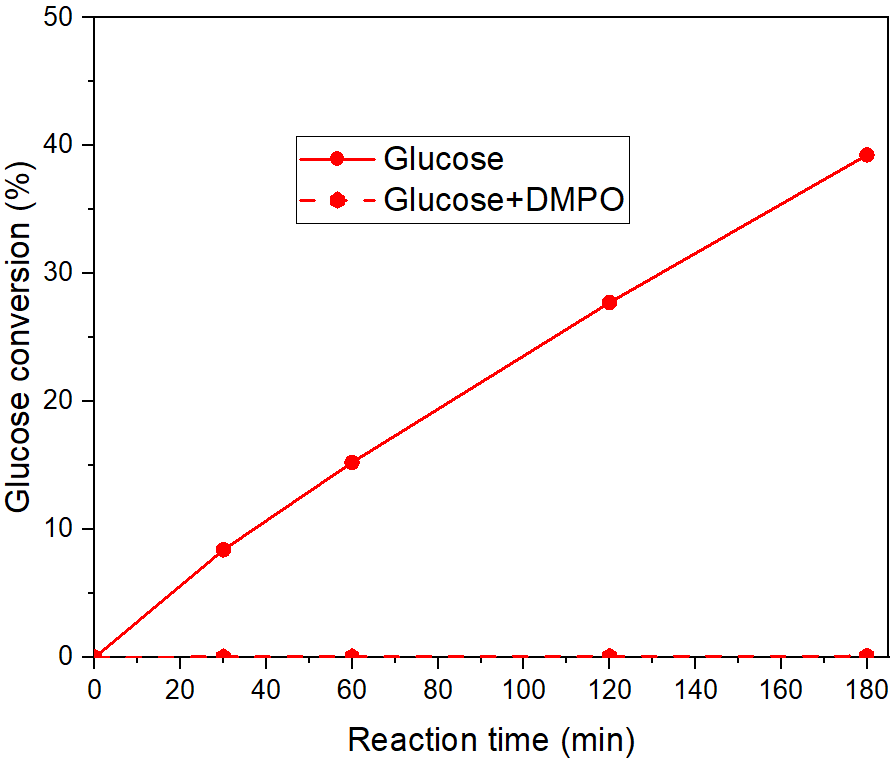


**Figure S6.** Glucose conversion with (dashed line) and without DMPO (plain line). Reaction conditions: 1mM of Furfural, 5 mM of DMPO, 100 mL of H_2_O, 35 ^°^C, 545 kHz, Ar flow 30 mL/min.

**Table S1**: Real time analysis of the gas phase of the ultrasonic reactor. Conditions: Ar (30 mL/min), 35 °C, 545 kHz.

| Gas | H_2_ | CH_4_ | CO_2_ | C_2_H_4_+C_2_H_2_ |
| --- | --- | --- | --- | --- |
| Amount in ppm | 1383^[a]^ | 437 | 206 | 120 |

[a] 41% stems from water sonolysis and 59% from impurities thermal cracking

**Online analysis of the gas phase by gas chromatography**: The gaseous products formed during the ultrasonic irradiation of aqueous glucose solution containing impurities were monitored online using a Micro GC Fusion Gas Analyzer (INFICON) connected to the gas outlet of the ultrasonic reactor. Gas sampling was performed directly at the reactor outlet through a heated injection loop (90 °C) to prevent condensation. The GC was equipped with two capillary columns mounted in parallel, allowing for simultaneous and complementary separation of the various gases. The first column, Rt-Msieve 5A (30 m, 0.53 mm ID, 50 µm; column temperature 80 °C; carrier gas: Ar), was used for H₂, O₂, N₂, CH₄, and CO, while the second column, Rt-Q-BOND (30 m, 0.53 mm ID, 20 µm; temperature programmed from 50 to 160 °C at 1 °C·s⁻¹; carrier gas: He), was used for CH₄, CO₂, light hydrocarbons, and H₂S. Detection was performed using a thermal conductivity detector (TCD) at 70 °C. This setup enabled on line identification and quantification of the main gaseous products (H₂, CO, CO₂, CH₄, etc.).

**Figure S7**.*^1^H* NMR (D_2_O, 500MHz) of glucose (1mM) and its impurities at T0 and after 2 h of ultrasonic irradiation. Reaction conditions: carried out in 50mL D_2_O, 35 ^°^C, 545 kHz, Ar (30 mL/min).

Table S2. Few oxygenated products detected in trace amount by LC-HRMS

| Molecular formula | Theoretical mass [M-H]^+^ | Found Experimentally [M-H]^+^ |
| --- | --- | --- |
| C_3_H_6_O_3_ | 91.0390 | 91.0392 |
| C_4_H_4_O_2_ | 85.0284 | 85.0287 |
| C_5_H_4_O_3_ | 113.0233 | 113.0223 |
| C_6_H_10_O | 99.0804 | 99.0806 |

Under Ar after 4 h

Formic acid

**Figure S8**. HPLC chromatogram profiles collected during the ultrasonic treatment of glucose (1 mM) containing guaiacol, furfural and HMF as impurities (1 mM) under Ar atmosphere. T0 in blue and T0+4h in red. Reaction conditions: 100 mL of H_2_O, 35 ^°^C, 545 kHz, 4 h, Ar (30 mL/min).


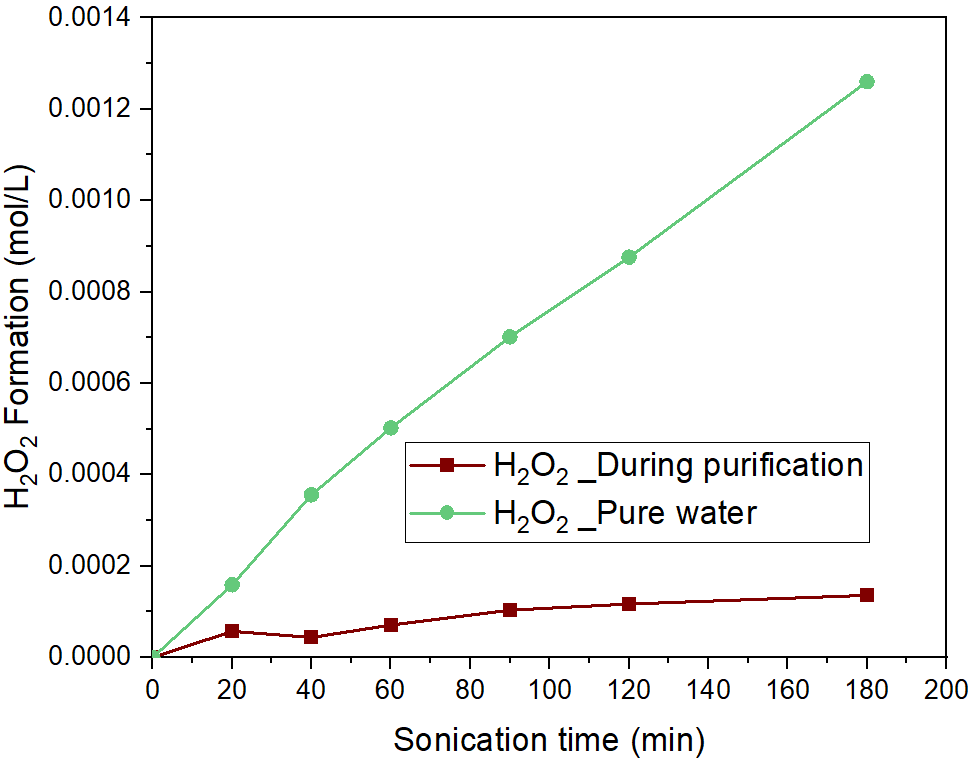


**Figure S9**. H_2_O_2_ formation in pure water (green) and in an aqueous solution containing guaiacol (1mM), furfural (1mM), HMF (1mM), and glucose (1mM). Reaction conditions: 100 mL of H_2_O or aqueous solution, 35 ^°^C, 545 kHz, Ar 30 mL/min.

**Figure S10** H_2_O_2_ formation as a function of the ultrasonic irradiation time under Ar (Orange) and Ar/O_2_ (green) flow. Reaction conditions: 100 mL H_2_O, 35 °C, 545 kHz, Ar and Ar/O_2_ flow 30 mL/min.

**Figure S11**. HPLC chromatogram profiles collected during the ultrasonic treatment of glucose (1 mM) containing guaiacol, furfural and HMF as impurities (1 mM each) under Ar/O_2_ atmosphere. Reaction conditions: 100 mL of H_2_O, 35 ^°^C, 545 kHz, 4 h, Ar (30 mL/min).

**LogP**

**Time required to reach 40% conversion (%)**

**Figure S12**. Plot of the LogP against the time required to reach 40% conversion for 6 impurities (in blue ●: 2-ethoxycatechol, syringol, guaiacol, vanillin, furfural and HMF), 5 impurities (in yellow ●: 2-ethoxycatechol, guaiacol, vanillin, furfural and HMF), 4 impurities (in grey ●: 2-ethoxycatechol, syringol, guaiacol, and HMF) and 3 impurities (in orange ●: guaiacol, furfural and HMF). Reaction conditions: Ar flow-30 mL/min, 35° C, 545kHz, 1mM each.


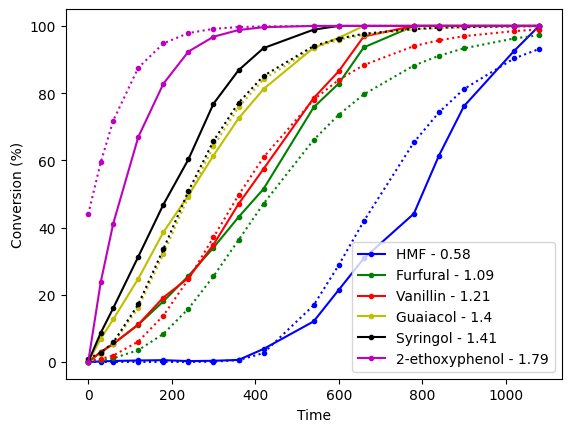


**Figure S13.** Comparison between experimental (plain lines) and modelized (dashed lines, Gompertz model) kinetic profile

The mathematical equation, describing the collected experimental kinetic profile, was derived by fitting a Gompertz model to each of the curves, using the programming language Python and the library Scipy. We added a time shift to model the difference in the order of reactivity. The Gompertz model can be expressed as such:

$$x=Ke^{-e^{-a(t-b)}}$$

where K, a and b are variables depending on the log P only, and x is the conversion rate. K is automatically equal to 100 as it represents the final value of the curve. When fitting a Gompertz model to each of the six curves independently, and without any constraints on a and b, the derived curves closely match the experimental curves, validating our model choice. We then expressed a and b as functions of log P; we chose to model a as a quadratic function of log P, and b as a linear function of log P. Therefore, the final model expression is:

$x=Ke^{be^{-at}}$, with $a=1.36.{10}^{-2}*logP^{2}-2.47.{10}^{-2}*logP+1.57.{10}^{-2}$ and $b= -537*logP+948$.


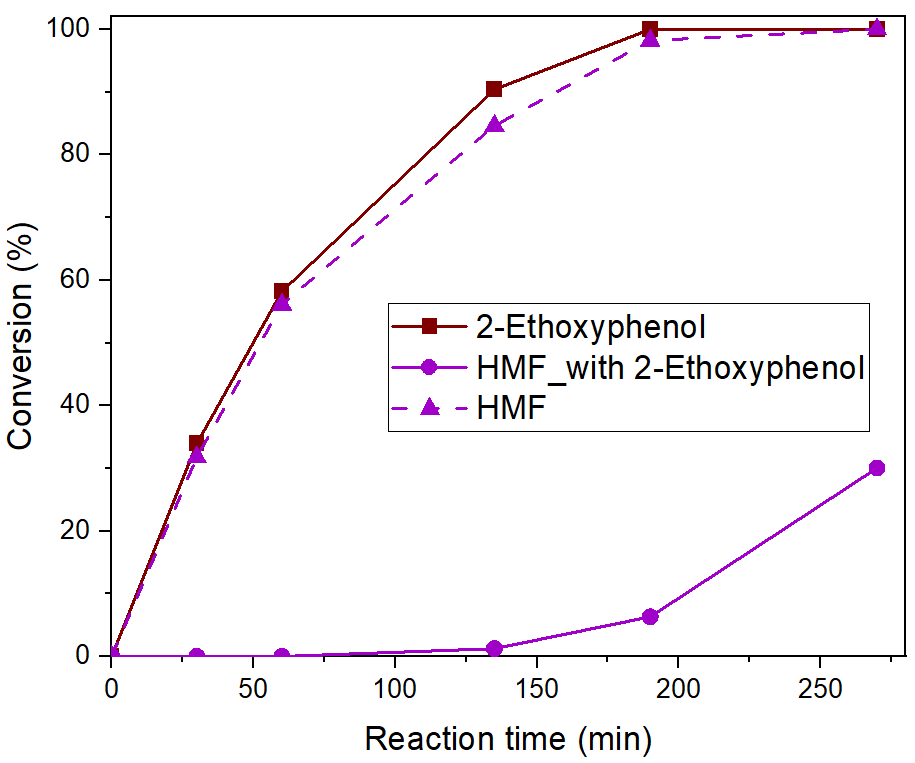


**Figure S14.** Ultrasonic irradiation of HMF with (plain line) and without 2-ethoxyphenol (dashed line). Reaction conditions: 1 mM of 2-ethoxyphenol and 1mM HMF, 100 mL of H_2_O, 35 ^°^C, 545 kHz, Ar flow 30 mL/min.


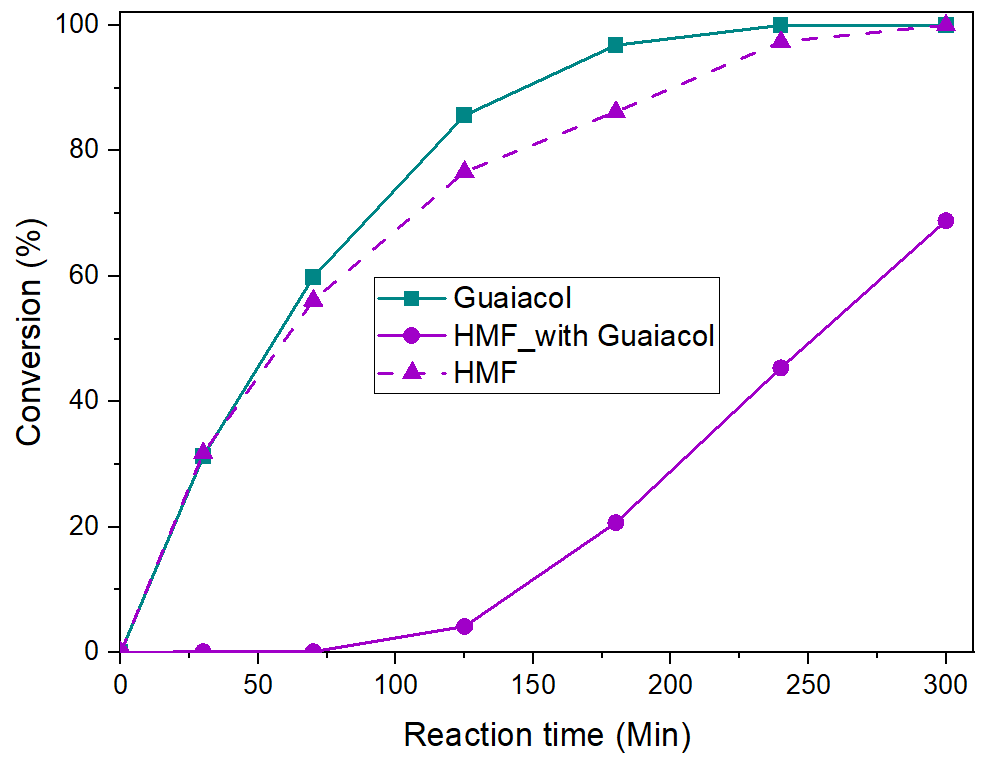


**Figure S15.** Ultrasonic irradiation of HMF with (plain line) and without guaiacol (dashed line). Reaction conditions: 1 mM of guaiacol and 1 mM HMF, 100mL of H_2_O, 35 ^°^C, 545 kHz, Ar flow 30 mL/min.

**Fig. S16.** Ultrasonic irradiation of 2-ethoxyphenol (A) 1 mM HMF and (B) 10 mM HMF. Reaction conditions: 1 mM of 2-ethoxyphenol and 1-10 mM HMF, 100 mL of H_2_O, 35 °C, 545 kHz, Ar flow 30 mL/min


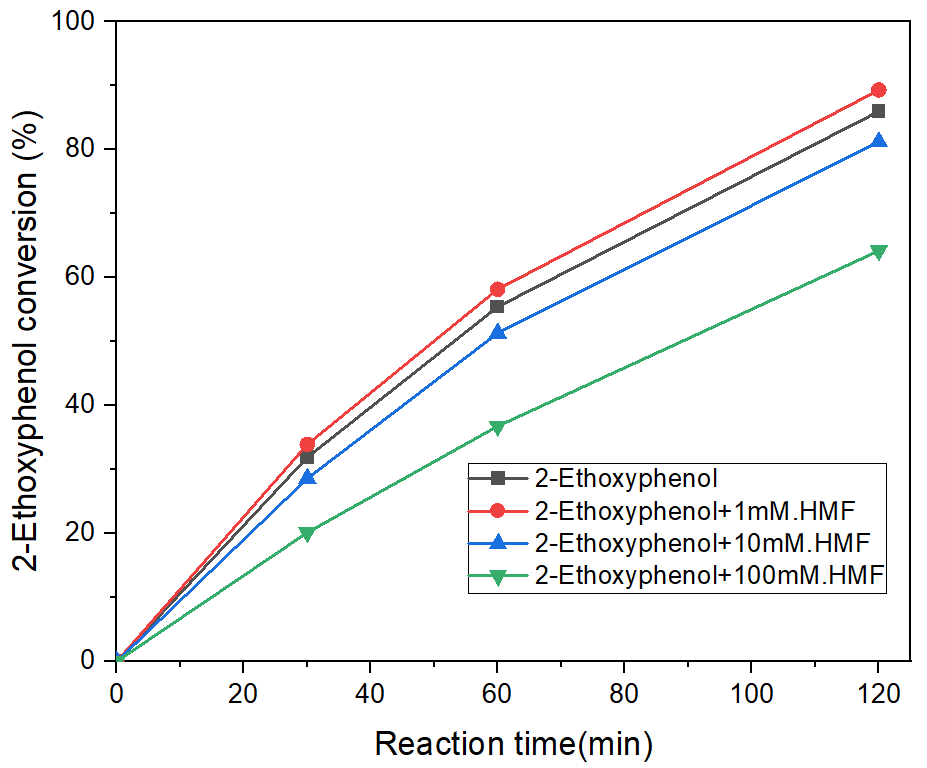


**Figure S17**. 1mM 2-ethoxyphenol conversion as a function of the ultrasonic treatment time in the presence of different concentrations of HMF (1-100 mM). Reaction conditions: 100 mL of H_2_O, 35 ^°^C, 545 kHz, Ar flow 30 mL/min.


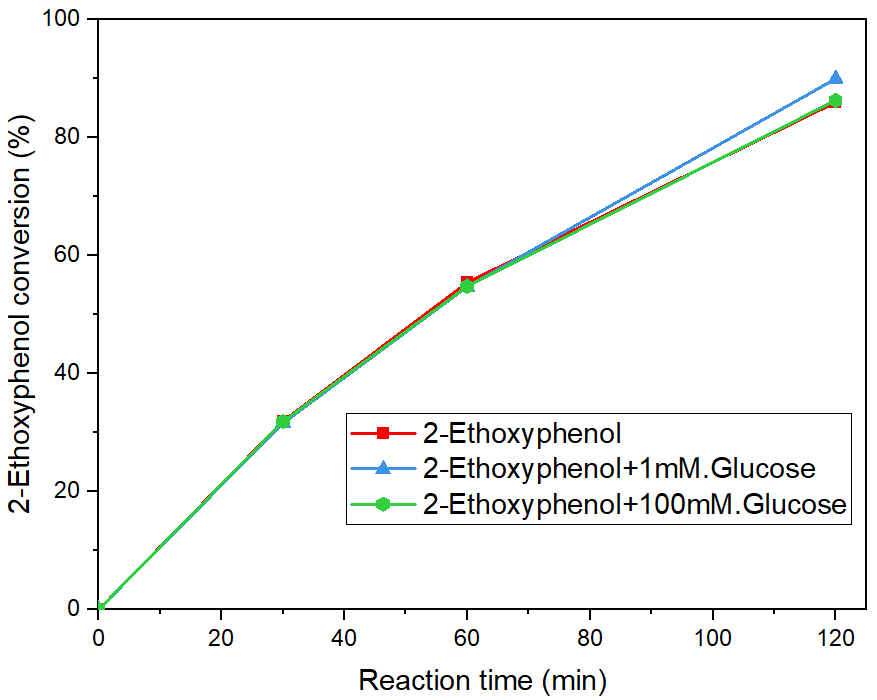


**Figure S18**. 1mM 2-ethoxyphenol conversion as a function of the ultrasonic treatment time in the presence of different concentrations of glucose (1-100 mM). Reaction conditions: 100 mL of H_2_O, 35 ^°^C, 545 kHz, Ar flow 30 mL/min.


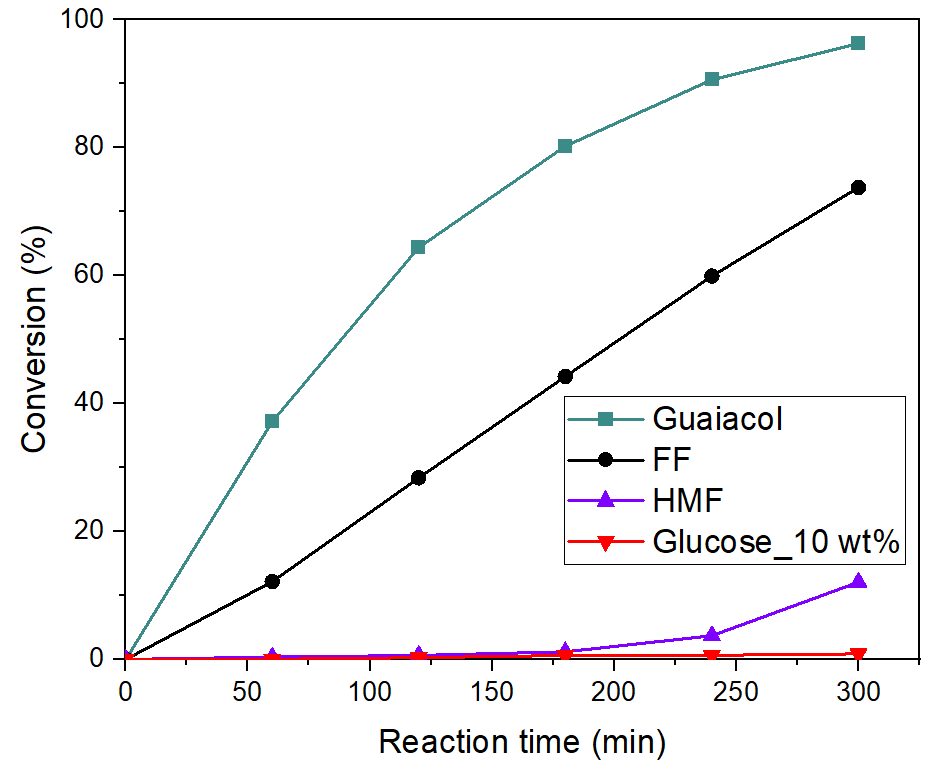


**Figure S19.** Removal of impurities composed of 1 mM guaiacol, 1 mM furfural and 1 mM HMF from 10 wt% aqueous glucose syrup. Reaction conditions: 100 mL of H_2_O, 35 °C, 545 kHz, Ar flow 30 mL/min.

**Table S3**. Conversion rate of impurities (furfural, HMF and guaiacol) as a function of the glucose concentration. Reaction conditions: 100 mL of H_2_O, 35 °C, 545 kHz, Ar flow 30mL/min

| Impurity | Glucose concentration | | |
| --- | --- | --- | --- |
|  | 1mM | 5mM | 10%w |
| Conv. rate furfural | 1.78 x10^-4^mol/L/h | 1.29 x10^-4^mol/L/h | 1.26 x10^-4^ mol/L/h |
| Conv. rate Guaiacol | 4.86 x10^-4^ mol/L/h | 4.51 x10^-4^mol/L/h | 4.32 x10^-4^ mol/L/h |
| Conv. rate HMF | 1.54x10^-4^ mol/L/h | 1.63 x10^-4^ mol/L/h | 1.29x10^-4^ mol/L/h |


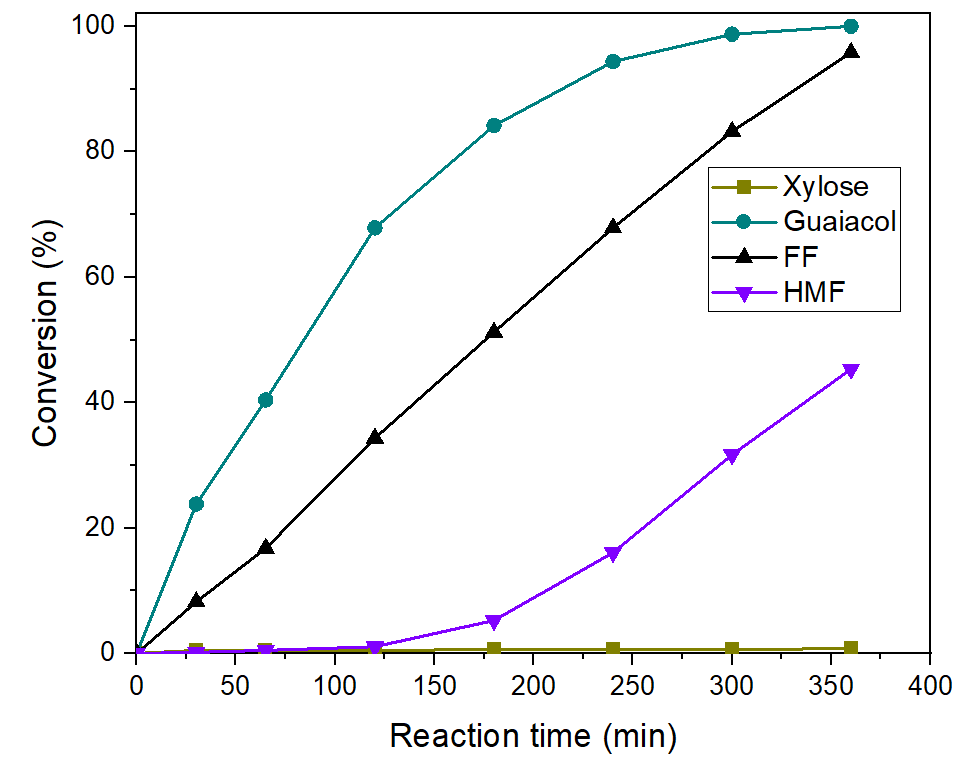


**Figure S20**. Removal of impurities composed of 1 mM guaiacol, 1 mM furfural and 1 mM HMF from 1 mM aqueous xylose solution. Reaction conditions: 100 mL of H_2_O, 35 °C, 545 kHz, Ar flow 30 mL/min.


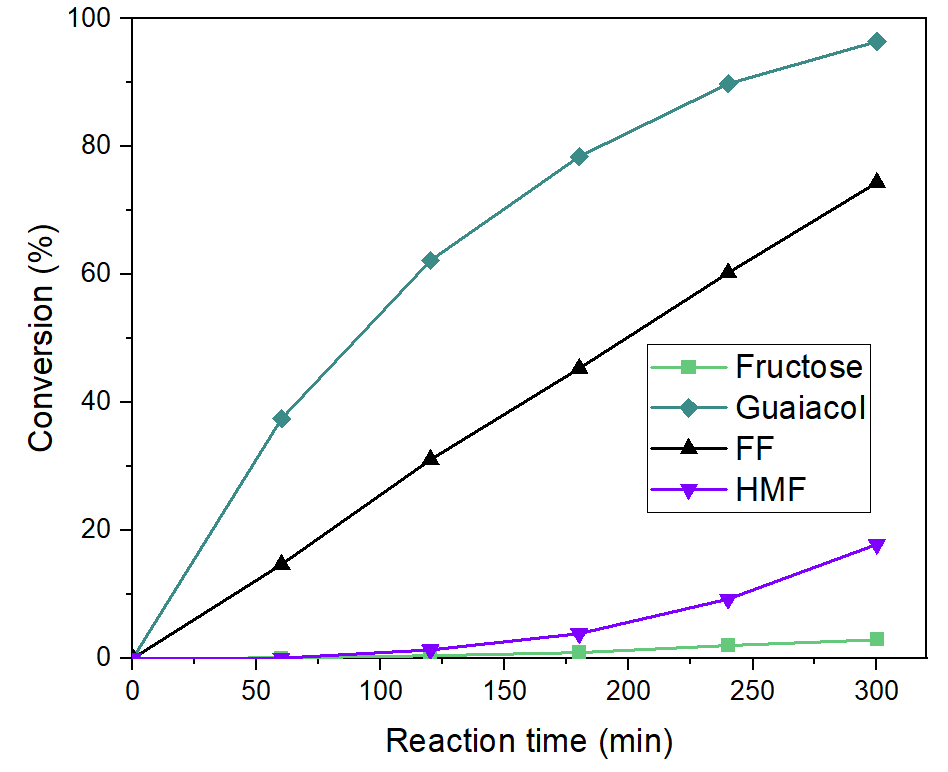


**Figure S21**. Removal of impurities composed of 1 mM guaiacol, 1 mM furfural and 1 mM HMF from 1 mM aqueous fructose solution. Reaction conditions: 100 mL of H_2_O, 35 °C, 545 kHz, Ar flow 30 mL/min.


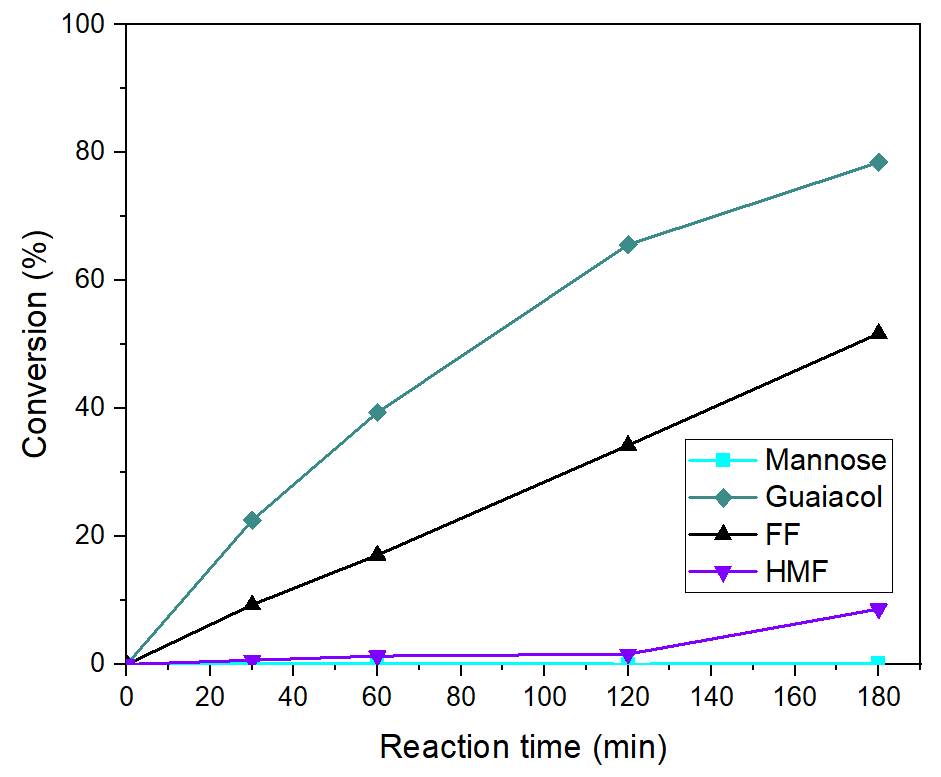


**Figure S22**. Removal of impurities composed of 1 mM guaiacol, 1 mM furfural and 1 mM HMF from 1 mM aqueous mannose solution. Reaction conditions: 100 mL of H_2_O, 35 °C, 545 kHz, Ar flow 30 mL/min.


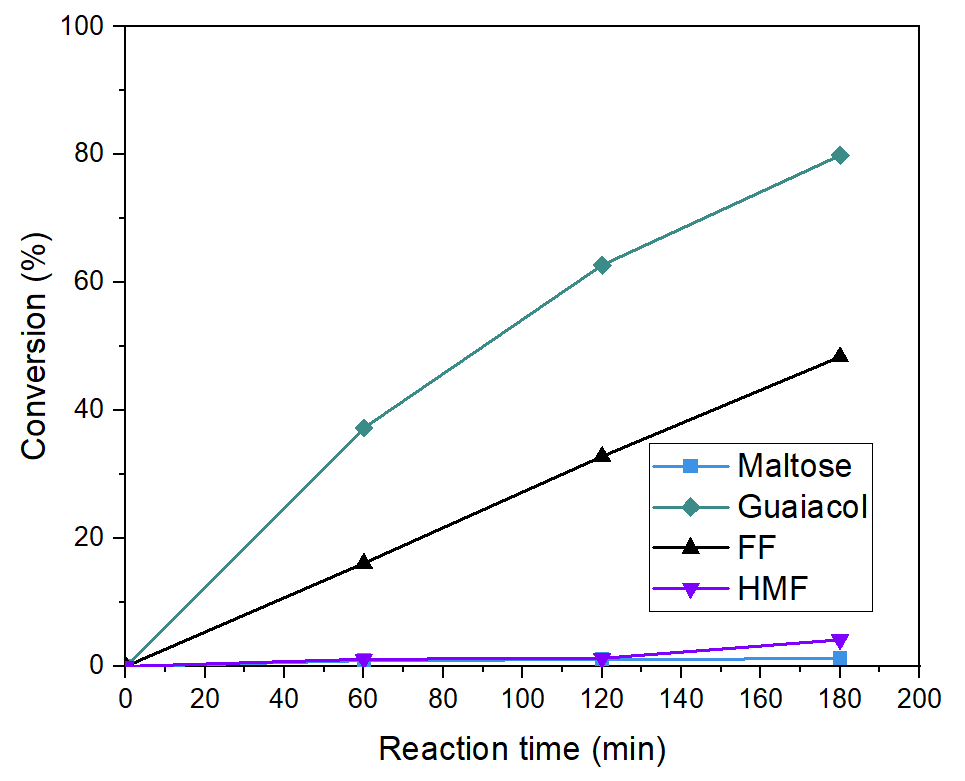


**Figure S23**. Removal of impurities composed of 1 mM guaiacol, 1 mM furfural and 1 mM HMF from 1 mM aqueous maltose solution. Reaction conditions: 100 mL of H_2_O, 35 °C, 545 kHz, Ar flow 30 mL/min.


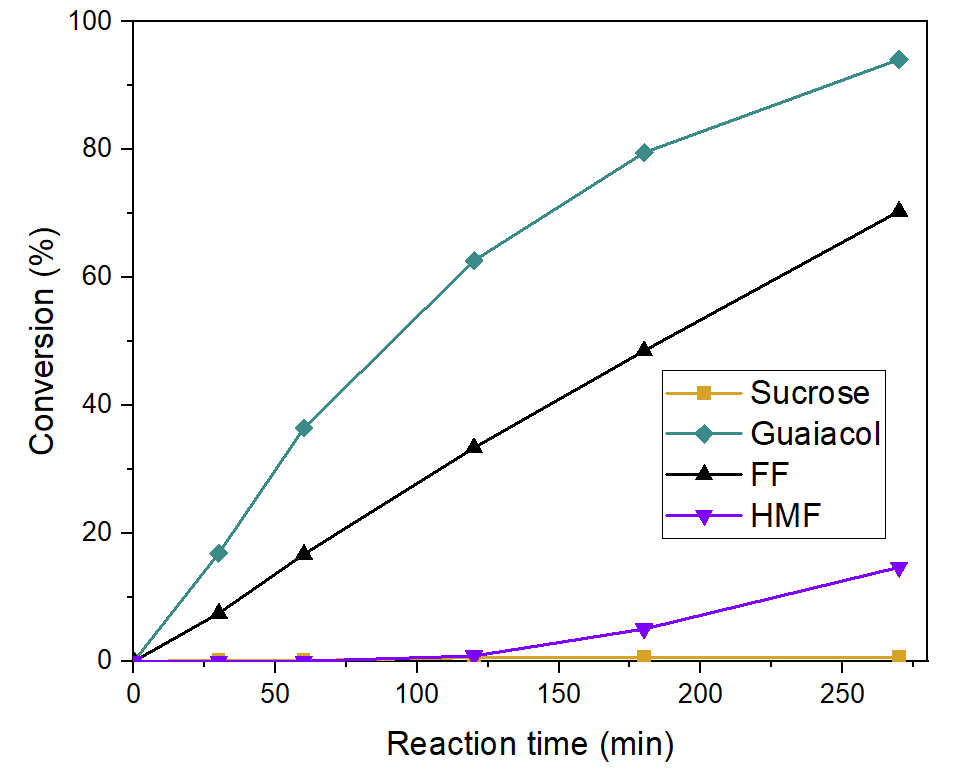


**Figure S24**. Removal of impurities composed of 1 mM guaiacol, 1 mM furfural and 1 mM HMF from 1 mM aqueous sucrose solution. Reaction conditions: 100 mL of H_2_O, 35 °C, 545 kHz, Ar flow 30 mL/min.


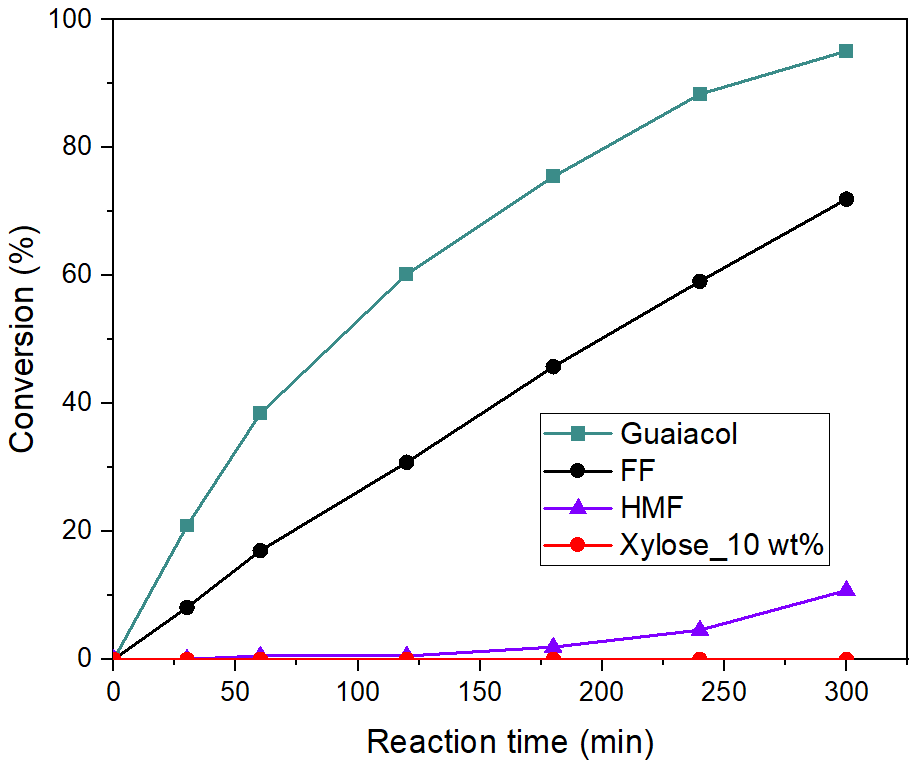


**Fig. S25**. Elimination of impurities consisting of 1 mM guaiacol, 1 mM furfural, and 1 mM HMF from a 10 wt% aqueous xylose solution. Reaction conditions: 100 mL of H_2_O, 35 °C, 545 kHz, Ar flow 30 mL/min.


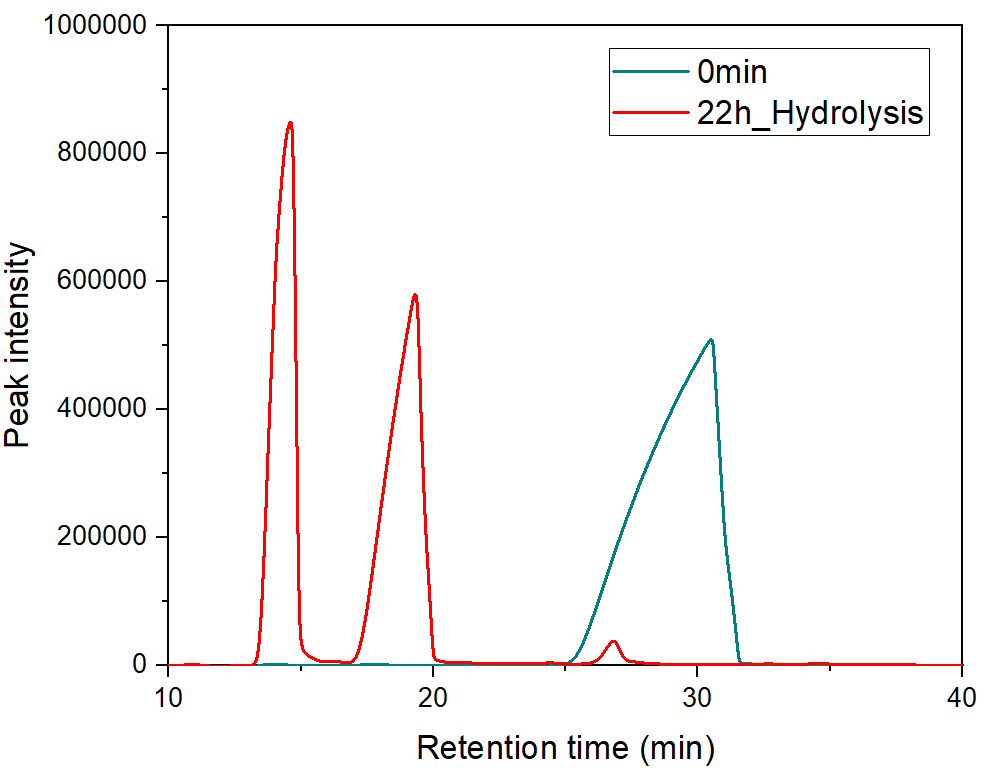


Sucrose

Fructose

Glucose

**Figure S26**. HPLC chromatogram collected during the hydrolysis of 30 wt% sucrose to glucose and fructose. Reaction conditions: Aquivion® PFSA (2 mol% H^+^), 100 mL H_2_O and at 60 °C.


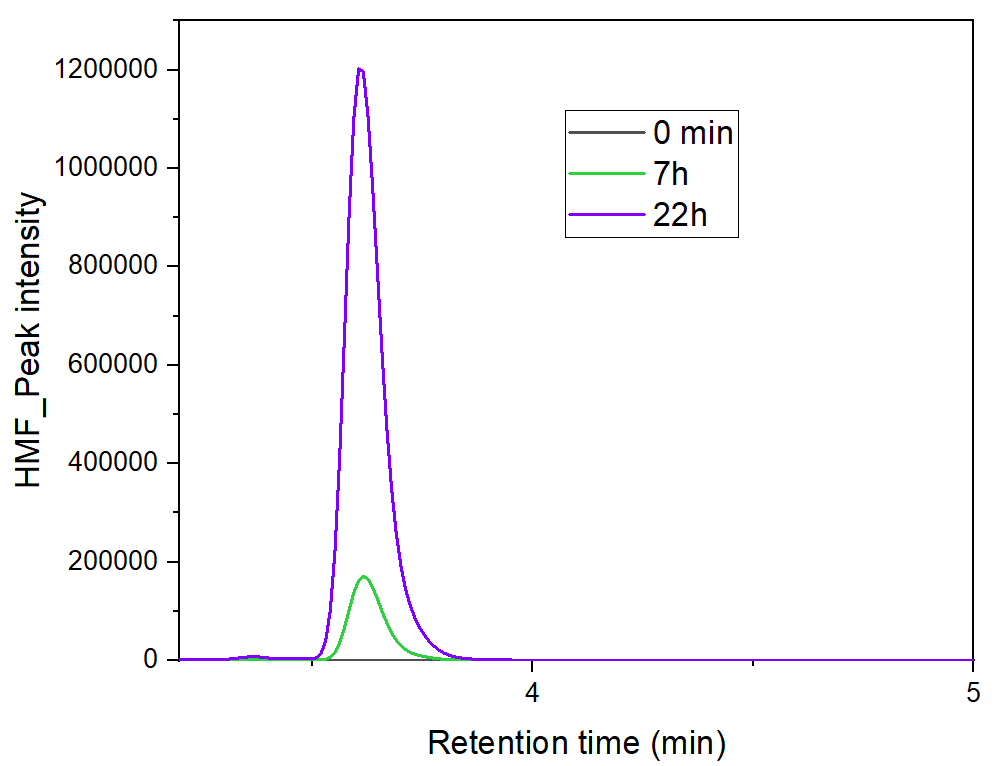


**Figure S27**. HPLC chromatogram showing the accumulation of HMF as a co-product during the hydrolysis of 30 wt% sucrose to glucose and fructose. Reaction conditions: Aquivion® PFSA (2 mol% H^+^), 100 mL H_2_O and at 60 °C.


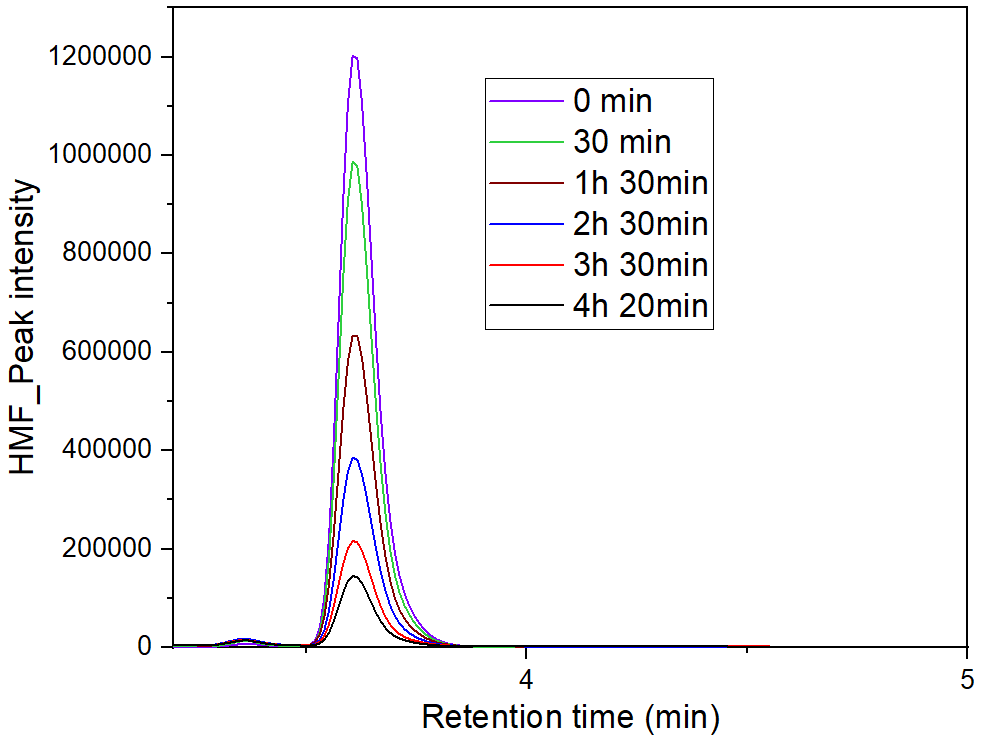


**Figure S28**. HPLC monitoring of HMF removal from a sucrose hydrolysate using high-frequency ultrasound. Reaction conditions: 100 mL of H_2_O, 35 °C, 545 kHz, Ar flow 30 mL/min.


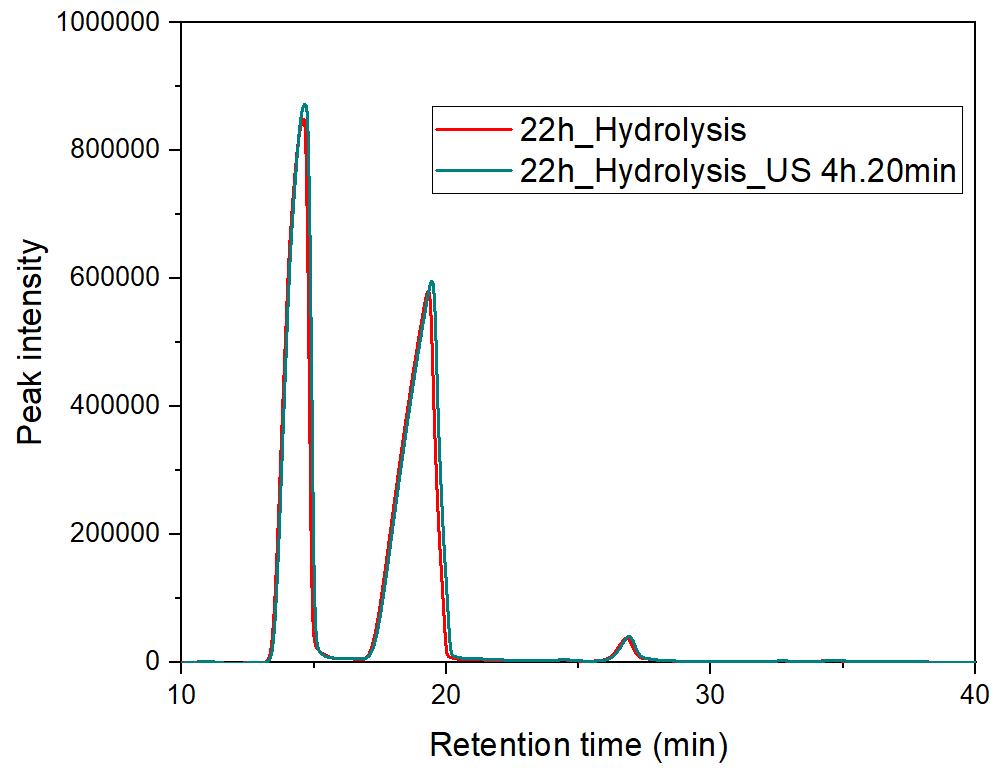


Sucrose

Glucose

Fructose

**Figure S29**. HPLC chromatogram collected during the removal of HMF from sucrose hydrolysate and showing that glucose and fructose remains unreacted. Reaction conditions: 100 mL of H_2_O, 35 °C, 545 kHz, Ar flow 30 mL/min.
